# A compendium of uniformly processed human gene expression and splicing quantitative trait loci

**DOI:** 10.1038/s41588-021-00924-w

**Published:** 2021-09-06

**Authors:** Nurlan Kerimov, James D. Hayhurst, Kateryna Peikova, Jonathan R. Manning, Peter Walter, Liis Kolberg, Marija Samoviča, Manoj Pandian Sakthivel, Ivan Kuzmin, Stephen J. Trevanion, Tony Burdett, Simon Jupp, Helen Parkinson, Irene Papatheodorou, Andrew D. Yates, Daniel R. Zerbino, Kaur Alasoo

**Affiliations:** 1 Institute of Computer Science, University of Tartu, Tartu, Estonia; 2 Open Targets, Wellcome Genome Campus, Cambridge, UK; 3 European Molecular Biology Laboratory, European Bioinformatics Institute, Cambridge, UK

**Keywords:** Transcriptomics, Genetic association study, Gene expression

## Abstract

Many gene expression quantitative trait locus (eQTL) studies have published their summary statistics, which can be used to gain insight into complex human traits by downstream analyses, such as fine mapping and co-localization. However, technical differences between these datasets are a barrier to their widespread use. Consequently, target genes for most genome-wide association study (GWAS) signals have still not been identified. In the present study, we present the eQTL Catalogue (https://www.ebi.ac.uk/eqtl), a resource of quality-controlled, uniformly re-computed gene expression and splicing QTLs from 21 studies. We find that, for matching cell types and tissues, the eQTL effect sizes are highly reproducible between studies. Although most QTLs were shared between most bulk tissues, we identified a greater diversity of cell-type-specific QTLs from purified cell types, a subset of which also manifested as new disease co-localizations. Our summary statistics are freely available to enable the systematic interpretation of human GWAS associations across many cell types and tissues.

## Abstract (web summary)

The eQTL Catalogue provides uniform processing of 21 eQTL studies, allowing the identification of highly reproducible eQTLs affecting whole gene and transcript expression levels.

## Main

Gene expression and splicing QTLs are a powerful tool to link disease-associated genetic variants to putative target genes. However, despite efforts by large-scale consortia such as GTEx^1^ and eQTLGen^2^ to provide comprehensive eQTL annotations for a large number of human tissues, target genes and relevant biological contexts for most GWAS signals have not been found yet. Systematic co-localization efforts based on GTEx data have identified putative target genes for 47% of the GWAS loci^3^. Still, these genetic effects mediate only 11% of disease heritability^4^, suggesting that many regulatory effects cannot be detected in bulk tissues at a steady state^5^. In contrast, profiling specialized disease-relevant cell types, such as induced pluripotent stem cells^6^, peripheral immune cells^7^, microglia^8,9^ or dopaminergic neurons^10^, often identifies additional co-localizations that are missing in GTEx. Although several databases have been developed to collect eQTL summary statistics from individual studies^11–17^, these efforts have relied on the heterogeneous set of files provided by the original authors. These results often contain only a small subset of significant associations or lack essential details such as effect alleles, standard errors or sample sizes, which limit the downstream co-localization and Mendelian randomization analyses that can be performed^18^.

Moreover, there is considerable technical variation between studies in sample collection, RNA-sequencing (RNA-seq) protocols, genotyping and data analysis. Thus, it is currently unclear how strongly eQTL effect sizes are influenced by technical differences in sample collection, how many eQTLs are broadly shared, and what fraction is specific to a given cell or tissue type and could thus give rise to new disease co-localizations. Although analyses based on GTEx data have generally estimated high levels of eQTL sharing between most bulk tissues^1,19^, smaller studies have often estimated much lower levels of sharing between purified cell types^20,21^. However, these analyses are sensitive to how sharing is defined, which genes and variants are included in the analysis, and which analytical approaches are used^19,22^. Thus, it is impossible to directly compare the estimates of eQTL sharing between studies without reanalyzing the individual-level data with uniform methods.

Recent methodological advances have made it feasible to fine map genetic associations to small credible sets of putative causal variants and distinguish between multiple independent genetic signals in the region^23,24^. These fine-mapping results can be directly used in co-localization analysis^25^. They can also help avoid the many false-negative co-localizations missed by approaches that assume a single causal variant in the region of interest^18^. However, reliable fine mapping requires precise information about in-sample linkage disequilibrium (LD) between genetic variants which is usually not available^26,27^.

**Fig. 1:**
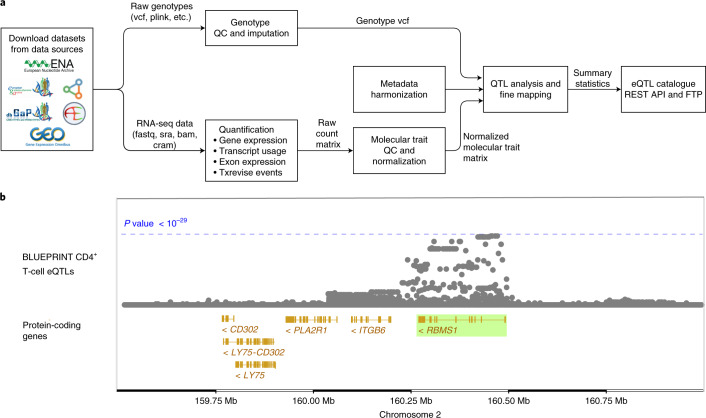
Overview of the eQTL Catalogue database. **a**, A high-level representation of the uniform data harmonization and eQTL mapping process. Extended Data Fig. 1 provides a schematic illustration of the different quantification methods. **b**, The eQTL Catalogue summary results for the *RBMS1* gene in BLUEPRINT CD4^+^ T cells, viewed via the Ensembl Genome Browser.

**Fig. 2:**
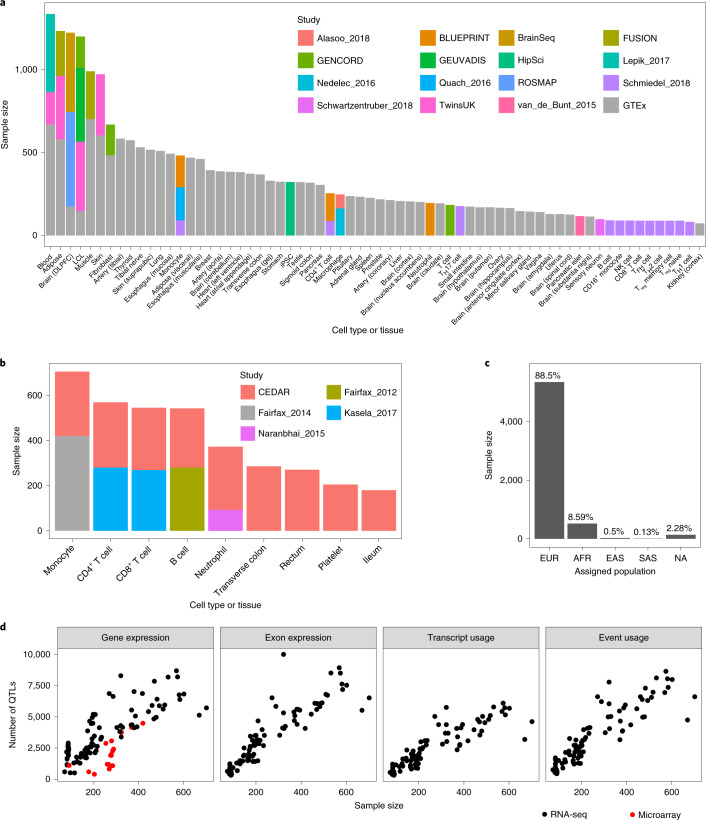
Overview of studies and samples included in the eQTL Catalogue. **a**, Cumulative RNA-seq sample size for each cell type and tissue across 16 studies. Datasets from stimulated conditions have been excluded to improve readability. DLPFC, dorsolateral prefrontal cortex; NK cell, natural killer cell; T_FH_ cell, follicular helper T cell; T_H_ cell, helper T cell; T_reg_, regulatory T cell. **b**, The cumulative microarray sample size for each cell type and tissue across five studies. Datasets from stimulated conditions have been excluded to improve readability. **c**, The number of unique donors assigned to the four major superpopulations in the 1000 Genomes phase 3 reference dataset. Detailed assignment of donors to the four superpopulations in each study is presented in Supplementary Table 1. Superpopulation codes: EUR, Europe; AFR, Africa; EAS, east Asia; SAS, south Asia; NA, unassigned. **d**, The relationship between the sample size of each dataset and the number of associations detected with each quantification method. The number of QTLs on the *y* axis is defined as the number of genes with at least one significant QTL (FDR < 0.05).

To overcome these limitations, we have uniformly re-processed (Fig. 1a) individual-level eQTL data from 112 datasets across 21 independent studies (see Fig. 2). We found that eQTL effect sizes from matched cell types or tissues were generally highly reproducible between studies. Using both eQTL sharing and matrix factorization approaches on fine-mapped eQTL signals, we found that differences in eQTL effect sizes between datasets are dominated by biological differences between cell types and tissues rather than technical differences in sample processing. Uniformly processed summary statistics provided us with a unique opportunity to characterize eQTL diversity across 69 distinct cell types and tissues. Consistent with previous analyses by the GTEx project, we found high levels of *cis*-eQTL sharing between most bulk tissues. In contrast, we found that a much smaller proportion of eQTLs is shared between purified cell types and bulk tissues, and between different cell types. This eQTL diversity also manifests itself at the level of disease co-localization, where we detect many novel co-localizations that are missed when analyzing GTEx data alone. Finally, in addition to gene expression QTLs, we identified QTLs at the levels of exon expression, transcript usage and splicing, which were often absent from the original studies. Our uniformly processed QTL summary statistics and fine-mapping results are available from the eQTL Catalogue FTP server and REST API, and they can also be explored using the Ensembl Genome Browser^28^ (Fig. 1b).

## Results

### Studies, datasets and samples included in the eQTL Catalogue

We downloaded raw gene expression and genotype data from 16 RNA-seq and 5 microarray studies from various repositories. The RNA-seq data consisted of 23,839 samples spanning 95 datasets (defined as distinct cell types, tissues or contexts in which eQTL analysis was performed separately). These 95 datasets originated from 66 distinct cell types and tissues and 10 stimulated conditions (Fig. 2a). Similarly, the 4,631 microarray samples spanned 17 datasets from 8 distinct cell types and tissues, and 3 stimulated conditions (Fig. 2b). Although most cell types and tissues were profiled only by 2 of the largest studies (GTEx^1^ and Schmiedel_2018 (ref. ^21^); Fig. 2a), 13 cell types or tissues were captured by multiple studies, allowing us to characterize both technical and biological variability between datasets and studies. The total number of unique donors across the studies was 5,714, of which 89% had predominantly European ancestries and only 9% had African or African–American ancestries, with other ancestries being rare (Fig. 2c and Supplementary Table 1). Thus, similar to most GWAS studies, published eQTL studies also suffer from a lack of genetic diversity^29^.

**Extended Data Fig. 1:**
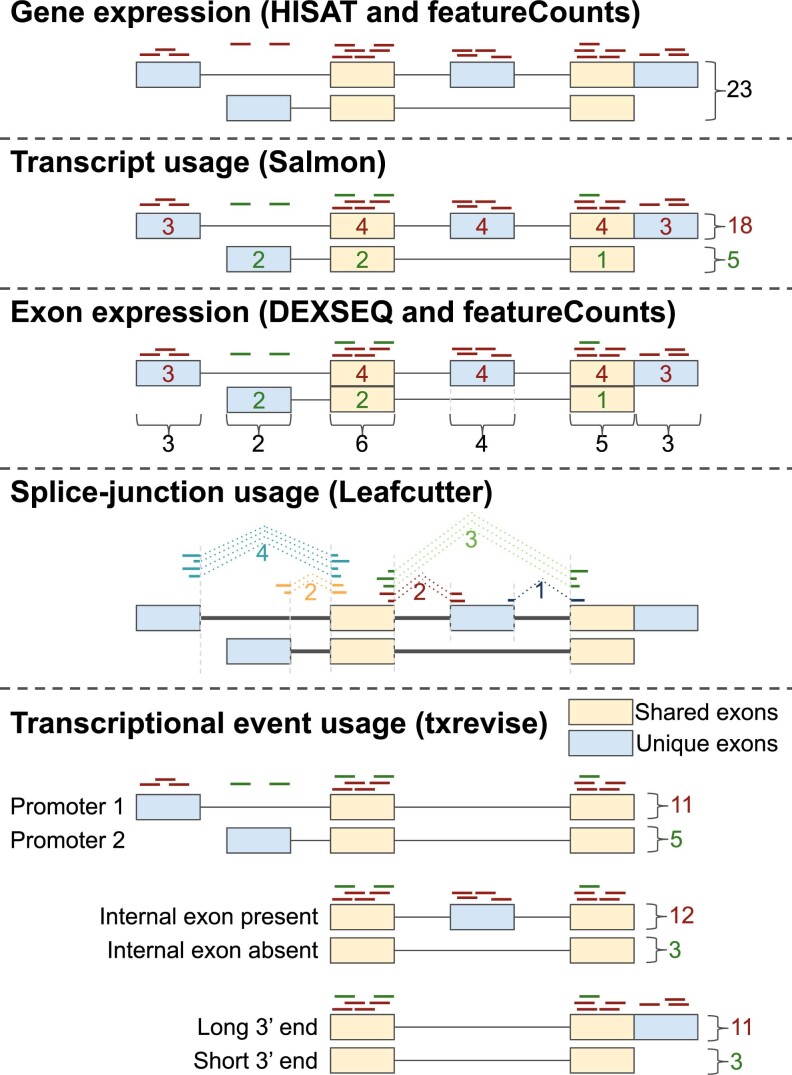
Quantification methods for molecular traits in the eQTL Catalogue. Symbolic representation of 23 read fragments assigned to one gene (aligned with HISAT2, quantified with featureCounts) consisting of two transcripts (quantified with Salmon) and six exonic parts (annotated with DEXSeq, quantified with featureCounts). The gene also has five distinct introns which are identified and quantified by Leafcutter. Transcriptional event usage is quantified with txrevise. Txrevise uses shared exons as a scaffold to identify independent transcriptional events corresponding to alternative promoters, internal exons and 3ʹ ends. Leafcutter splice junction QTLs will be included in a future version of the eQTL Catalogue.

To uniformly process a large number of eQTL studies, we designed a modular and robust data analysis workflow (Fig. 1a). First, we performed extensive quality control and imputed missing genotypes using the 1000 Genomes phase 3 reference panel^30^. For RNA-seq datasets, we performed QTL mapping for the four molecular traits described above (Fig. 1a and Extended Data Fig. 1). The QTL analysis was performed separately in each dataset (that is, separately for each cell type or tissue within each study). We found the largest number of QTLs at the level of gene expression, but for all molecular traits the number of significant associations scaled approximately linearly with the sample size (Fig. 2d and Supplementary Table 2). For microarray datasets, we performed the analysis only at the gene level but found the same linear trend (Fig. 2d and Supplementary Table 2). Our remaining analyses focused on the RNA-seq-based eQTL datasets because they covered a more comprehensive range of cell types and tissues, and accounted for most of the samples in the eQTL Catalogue.

### Biological and technical variability between studies

**Fig. 3:**
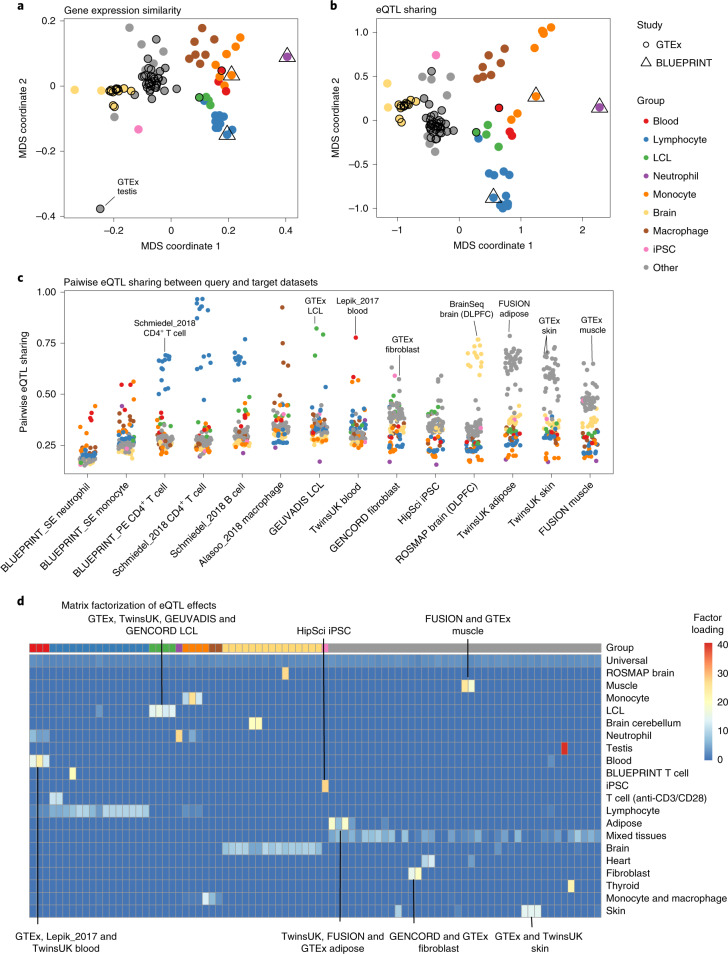
Gene expression similarity between datasets predicts eQTL similarity. **a**, MDS analysis of median gene expression across datasets. The pairwise similarity between datasets was calculated using Pearson’s correlation. Datasets from GTEx and BLUEPRINT studies have been highlighted to demonstrate that they cluster with other matching cell types and tissues. **b**, MDS analysis of eQTL sharing across datasets. Pairwise eQTL sharing between datasets was estimated using the Mash model. The complete matrix is presented in Extended Data Fig. 2. **c**, Visualization of eQTL-sharing estimates between selected representative tissues (*x* axis) and all other cell types and tissues in the eQTL Catalogue. The individual points have been colored according to the major cell type and tissue groups from **a**. **d**, Matrix factorization of the eQTL effect sizes across all eQTL Catalogue datasets. The heatmap represents the loadings of 21 latent factors in each of the 86 naive datasets. Nine datasets from stimulated macrophages and monocytes have been excluded to improve legibility. The version of this heatmap with dataset labels is shown in Extended Data Fig. 7.

First, we assessed whether the gene expression and eQTL signals were dominated by technical differences between studies (Supplementary Tables 3 and 4) rather than true biological differences between cell types and tissues. We visualized median transcripts per million (TPM) gene expression estimates from each dataset using multidimensional scaling (MDS). Reassuringly, we found that the datasets clustered predominantly by cell type or tissue of origin, rather than by studies or other technical factors (Fig. 3a). Notably, except for brain tissues, whole blood and testis, most other bulk tissues had relatively similar gene expression profiles (Fig. 3a). In contrast, datasets from purified cell types such as lymphoblastoid cell lines (LCLs), monocytes, neutrophils, induced pluripotent stem cells (iPSCs), and B and T lymphocytes had more distinct gene expression profiles (Fig. 3a).

Next, we performed the same similarity analysis on eQTL effect sizes. To overcome the high uncertainty associated with effect size estimates, especially in datasets with small sample sizes, we used the recently developed multivariate adaptive shrinkage (Mash) model^19^. Mash improves eQTL effect size estimates by sharing information across datasets as well as individual eQTLs. We limited our analysis to 62,837 fine-mapped eQTLs (see Methods) and defined two eQTLs to be shared between a pair of datasets if they had the same sign and their effect sizes did not differ more than twofold. We calculated pairwise eQTL-sharing estimates for all 95 RNA-seq datasets (including 49 tissues from GTEx) and projected those on to two dimensions using MDS. Consistent with previous reports by GTEx, we found that the eQTL similarity between datasets closely matched their gene expression similarity (Fig. 3a,b)^1^. This suggests that high gene expression similarity and a high degree of eQTL sharing both reflect similarity in the underlying regulatory state of cells.

**Extended Data Fig. 2:**
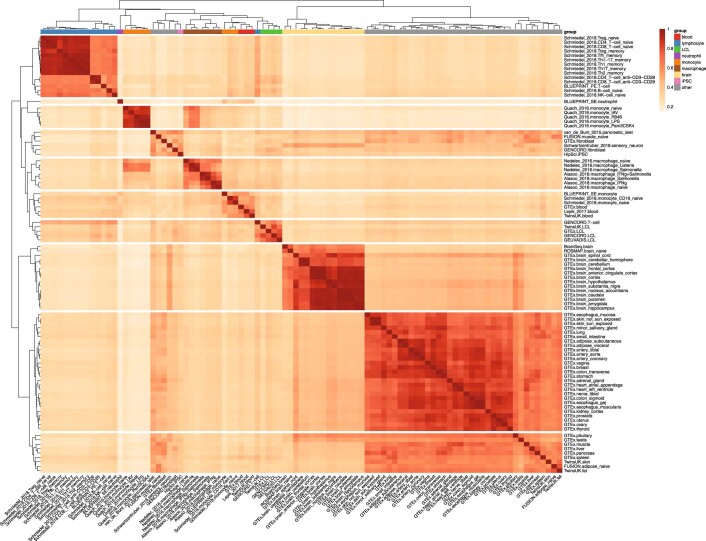
Pairwise eQTL sharing between 95 datasets estimated with the Mash model. We used 62,837 independent gene variant pairs from the fine mapping analysis (see Methods) and used the Mash model to estimate eQTL sharing between all pairs of the 95 datasets measured with RNA-seq. Heatmap represents the fraction of eQTLs ‘shared’ (same sign and effect size difference < 2-fold) between all pairs of datasets.

**Extended Data Fig. 3:**
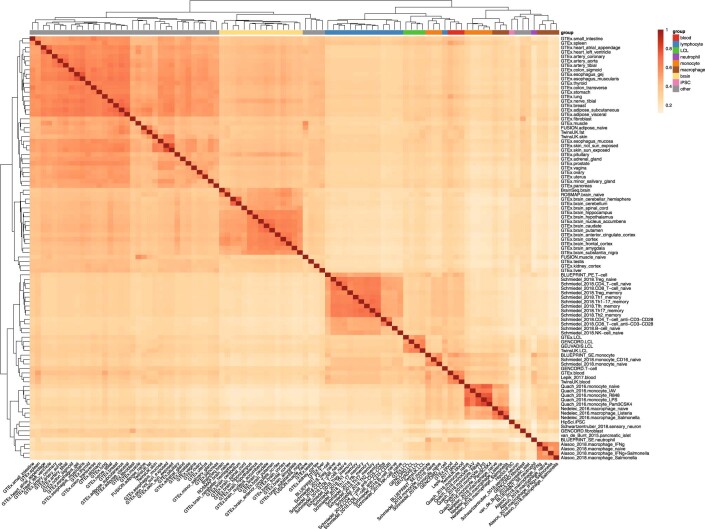
Pairwise eQTL similarity between 95 datasets estimated with Spearman correlation. We used 62,837 independent gene-variant pairs from the fine mapping analysis (see Methods) and used the Spearman correlation of eQTL effect sizes to estimate eQTL sharing between all pairs of the 95 datasets measured with RNA-seq. Heatmap represents the pairwise Spearman correlation estimates between fine mapped eQTL effect sizes.

**Extended Data Fig. 4:**
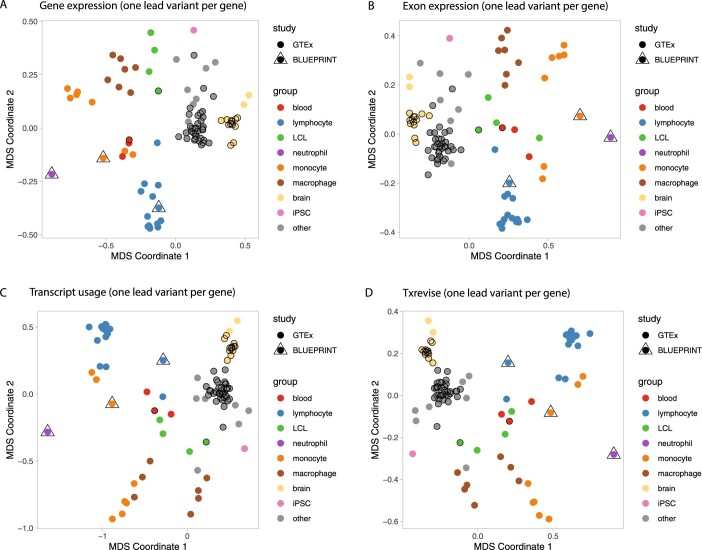
MDS analysis of QTL sharing across datasets. Pairwise QTL sharing between datasets was estimated using the Mash model. The individual points have been coloured according to the major cell type and tissue groups. To facilitate comparison between quantification methods, and avoid redundant signals from correlated transcripts and exons, all analyses have been performed using one lead variant per gene (see Methods). The panels show pairwise QTL sharing MDS plots for gene expression (**a**), exon expression (**b**), transcript usage (**c**), and txrevise (**d**) QTLs.

**Extended Data Fig. 5:**
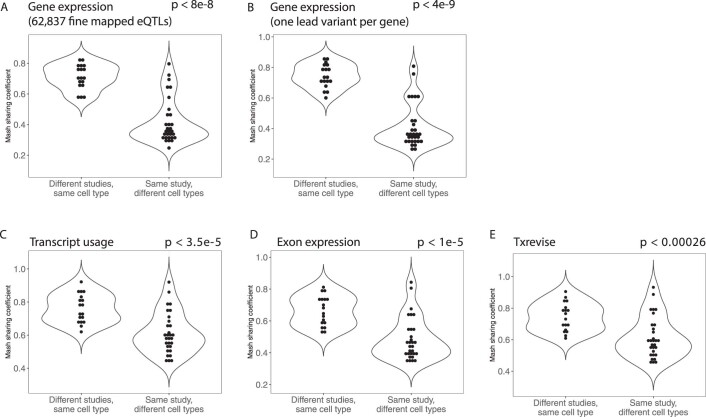
Quantifying QTL sharing between tissues, cell types and studies. Distribution of pairwise mash QTL sharing estimates for seven cell types and tissues (skin, adipose, LCL, blood, fibroblast, muscle, brain (DLPFC)) profiled in two or more studies (GTEx, TwinsUK, GENCORD, GEUVADIS, Lepik_2017, ROSMAP, FUSION). Each panel contrasts the QTL sharing estimates for the same cell type or tissue profiled in different studies (n = 18) against different cell types and tissues profiled in the same study (n = 30). Analysis was performed separately for gene expression (**a-b**), transcript usage (**c**), exon expression (**d**) and txrevise (**e**) QTLs. Note that adipose, skin and muscle tissues have high eQTL sharing also within the same study. The p-values were calculated using the two-sample Wilcoxon rank sum test (two-sided).

Next, we focused on potential batch effects between studies. Reassuringly, we found that, if the same cell type or tissue was profiled in multiple studies, then their eQTL effect sizes often showed a high degree of concordance (Fig. 3b and Extended Data Figs. 2 and 3). For example, LCLs from TwinsUK, GENCORD and GEUVADIS clustered together with LCLs from GTEx (Fig. 3b) and exhibited median sharing of ~80% (Fig. 3c and Extended Data Figs. 2 and 3). The same was also true for the brain (GTEx, ROSMAP and BrainSeq), whole blood (GTEx, TwinsUK and Lepik_2017), muscle (GTEx and FUSION), skin (GTEx and TwinsUK) and adipose tissues (GTEx, TwinsUK and FUSION), which all had median within-tissue sharing of ~70% (Fig. 3c). We also observed broadly similar patterns of sharing among the QTLs detected with the other three quantification methods (Extended Data Fig. 4). To assess this formally, we focused on a subset of tissues profiled in at least two studies. We found that the average eQTL sharing for the same tissue profiled in two different studies was significantly higher than for two different cell types or tissues profiled within the same study (Extended Data Fig. 5).

Finally, we focused on the patterns of sharing between different cell types and tissues. We found that 46–80% (median 62%) of the eQTLs were shared between most pairs of bulk tissues (Fig. 3c). The exceptions to this pattern were the brain tissues and whole blood that formed separate clusters in the MDS analysis (Fig. 3b) and shared a median of 45% and 35% of the eQTLs with other tissues, respectively (Fig. 3c). In contrast, purified immune cell types (LCLs, neutrophils, monocytes, macrophages and lymphocytes) formed distinct clusters on the MDS plot (Fig. 3b), and had much lower eQTL sharing with both whole blood and other bulk tissues (Fig. 3c). Thus, although our results reconfirm the generally high level of *cis*-eQTL sharing between bulk tissues, they also reveal a much greater *cis*-eQTL diversity between purified cell types and especially immune cells. Importantly, this diversity is missed when analyzing highly tissue-focused eQTL studies such as GTEx.

### Identification of tissue-specific and shared latent factors

**Extended Data Fig. 6:**
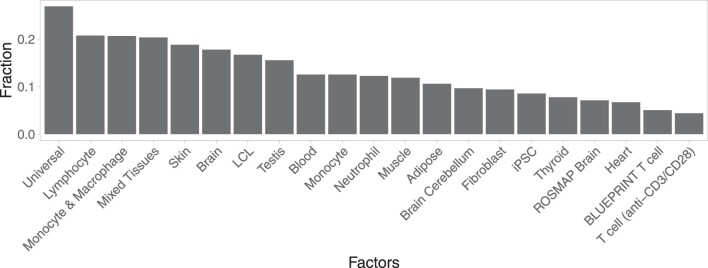
The fraction of fine mapped eQTLs assigned to universal and cell-type-specific factors. The sn-spMF method was used to assign all fine mapped eQTLs to the 21 latent factors inferred from the data.

**Extended Data Fig. 7:**
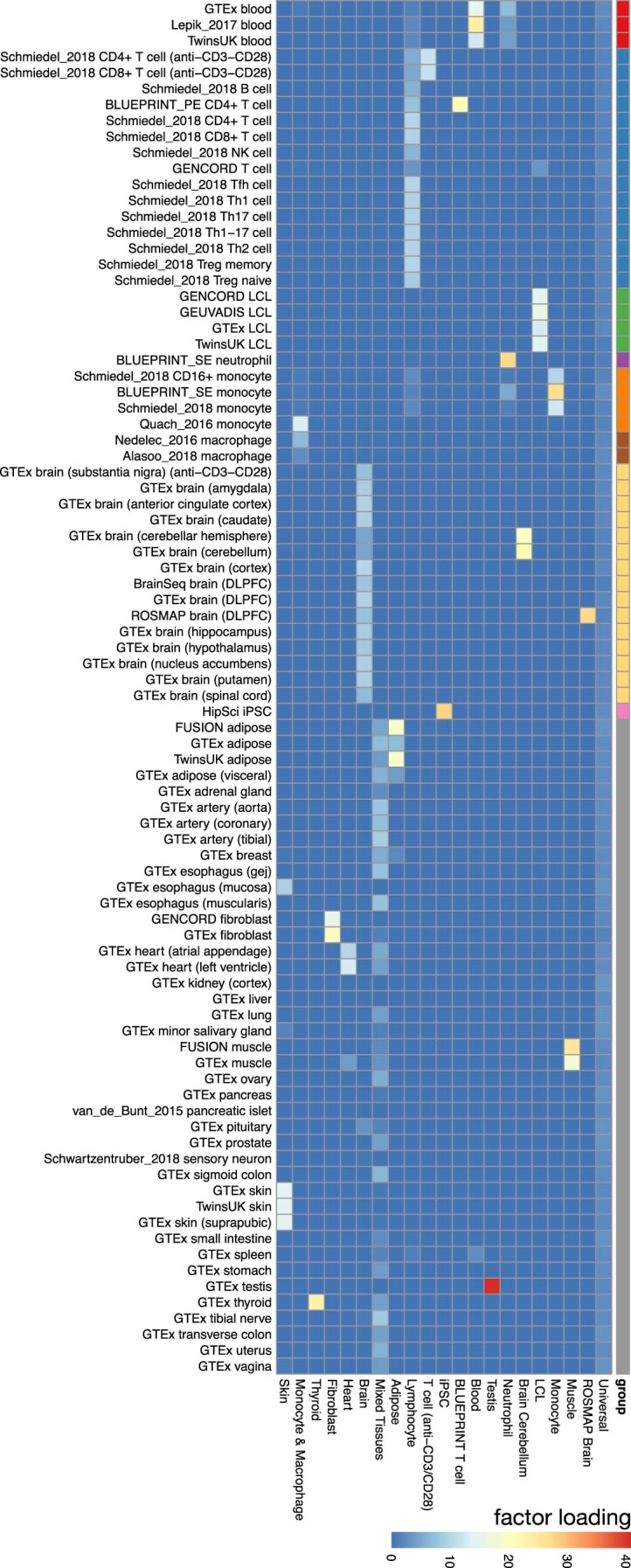
Factor loadings for each of the 86 naive datasets across 21 latent factors detected by sn-spMF. Datasets from stimulated monocytes and macrophages have been excluded to improve readability.

To better understand the eQTL-sharing patterns between cell types and tissues, we turned to a recently developed semi-non-negative sparse matrix factorization (sn-spMF) model that can directly identify latent factors from eQTL summary statistics^31^. When applied to the fine-mapped eQTL Catalogue summary statistics, sn-spMF detected 21 independent factors (Fig. 3d). The largest universal factor was broadly shared between all datasets and accounted for ~26.9% of the independent fine-mapped eQTLs (Extended Data Fig. 6). The remaining 20 factors captured cell-type- and tissue-specific effects (Fig. 3d and Extended Data Fig. 7). Overall, matrix factorization identified many of the same patterns detected in the pairwise eQTL-sharing analysis (Fig. 3b). For example, lymphocytes, LCLs, iPSCs, monocytes, macrophages, neutrophils and stimulated T cells, as well as brain and blood tissues all had their individual factors. Notably, these cell-type- and tissue-specific factors were shared across multiple studies (Fig. 3d).

Although most eQTLs were highly shared between bulk tissues (Fig. 3b,c), our factor analysis still detected independent factors capturing eQTLs that were specific to muscle, skin, testis, thyroid, heart and adipose tissues from the FUSION^32^, GTEx^1^ and TwinsUK^33^ studies. Together with brain and blood, these tissues had larger sample sizes than other bulk tissues and purified cell types (Fig. 2a), allowing us to obtain more accurate eQTL effect size estimates. Thus, we expect to detect additional tissue-specific factors as the sample sizes of the respective tissues increase^31^. Finally, only 2 of the 21 factors were specific to a single dataset (BLUEPRINT CD4^+^ T cells and ROSMAP brain samples), suggesting that, although batch effects between datasets exist, they are not a major factor confounding our analysis.

**Fig. 4:**
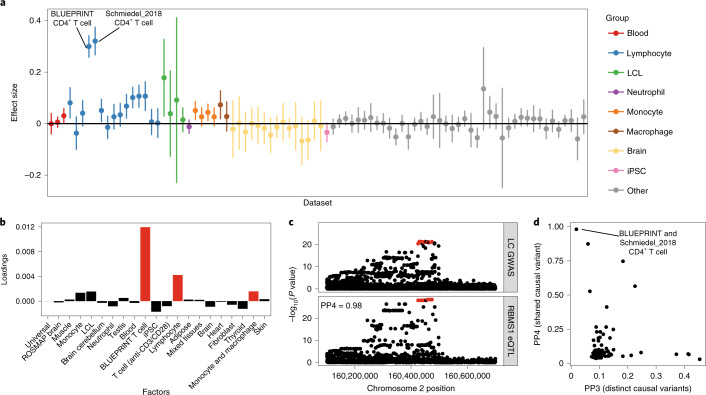
CD4^+^ T-cell-specific eQTL (rs7420451) at the *RBMS1* locus co-localizes with a GWAS hit for lymphocyte count. **a**, Variation in the *RBMS1* eQTL effect size across all eQTL Catalogue datasets (naive conditions only). The points represent the eQTL effect size estimates from the linear model, and the error bars represent 95% confidence intervals. Two CD4^+^ T-cell datasets (BLUEPRINT, *n* = 169; Schmiedel_2018, *n* = 88) have been highlighted. Sample sizes for other datasets are presented on Fig. 2a and in Supplementary Table 2. **b**, Factor loadings for the *RBMS1* lead variant (rs7420451) from the sn-spMF model. **c**, Regional association plot for lymphocyte count (top^34^) and *RBMS1* eQTL in the BLUEPRINT CD4^+^ T cells (bottom). The fine-mapped eQTL credible set is highlighted in red. **d**, Co-localization posterior probabilities between lymphocyte count and *RBMS1* expression in the region surrounding the *RBMS1* eQTL lead variant (rs7420451) across all eQTL Catalogue datasets. PP4 represents a shared causal variant whereas PP3 represents two distinct causal variants.

A major advantage of the matrix factorization is that it allows us to focus on a small number of biologically meaningful factors shared between one or more datasets rather than comparing the eQTL effect sizes in 95 individual datasets. This level of summarization is going to be increasingly important as the number of datasets included in the eQTL Catalogue increases. For example, a *cis*-eQTL for *RBMS1* had large effects in both BLUEPRINT and Schmiedel_2018 CD4^+^ T-cell datasets and smaller significant effects in multiple other T-cell subsets from Schmiedel et al.^21^ (Fig. 4a). Consequently, the two factors with the largest loadings for this eQTL were the BLUEPRINT CD4^+^ T-cell factor and the general lymphocyte factor (Fig. 4b). The *RBMS1* eQTL also co-localized with a GWAS signal for lymphocyte count^34^ in BLUEPRINT and Schmiedel_2018 CD4^+^ T cells (posterior probability 4 (PP4) >0.98; Fig. 4c), illustrating how a lymphocyte-specific eQTL might contribute to the regulation of lymphocyte count in whole blood. Notably, we did not detect this co-localization in any of the 49 GTEx tissues.

### Detection of additional co-localizations missed in GTEx

**Extended Data Fig. 8:**
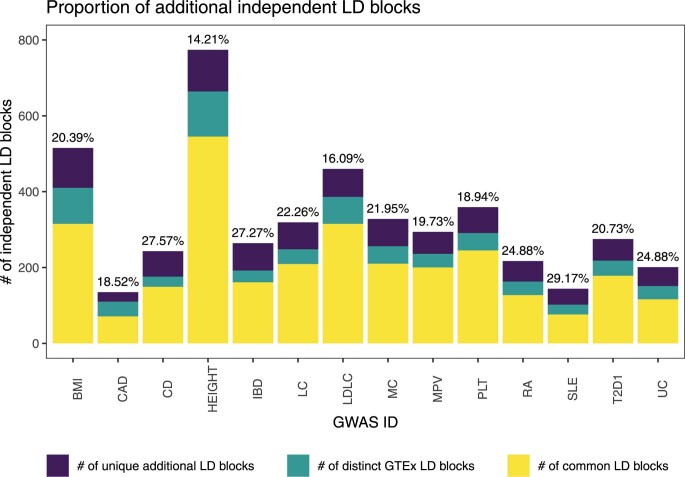
The number of shared and additional colocalisations detected for the 14 GWAS traits and diseases. The cumulative heights of the bars indicate the number of independent colocalising loci (LD blocks) detected for each GWAS trait and the percentages represent the fraction of those colocalisation that were unique to the eQTL Catalogue datasets relative to GTEx.

Our eQTL sharing analysis demonstrated that the eQTL Catalogue contains many additional eQTLs not present in GTEx. To quantify how these additional eQTLs might improve the interpretation of complex trait and disease associations, we performed co-localization between GWAS summary statistics for 14 traits and either the new eQTL Catalogue datasets or all GTEx tissues. To ensure that each independent GWAS locus was counted only once, we first partitioned GWAS summary statistics into approximately independent LD blocks^35^. Overall, we detected at least one co-localizing eQTL (PP4 ≥ 0.8) for 4,528 independent loci across 14 traits, 925 (20.4%) of which were detected in only one of the eQTL Catalogue datasets and not captured by GTEx (maximum PP4 < 0.8). The fraction of additional co-localizing loci varied from 14% for height to 29% for lupus (Extended Data Fig. 8), suggesting that a substantial fraction of trait co-localizations might be missed if the analysis were restricted only to GTEx.

**Fig. 5:**
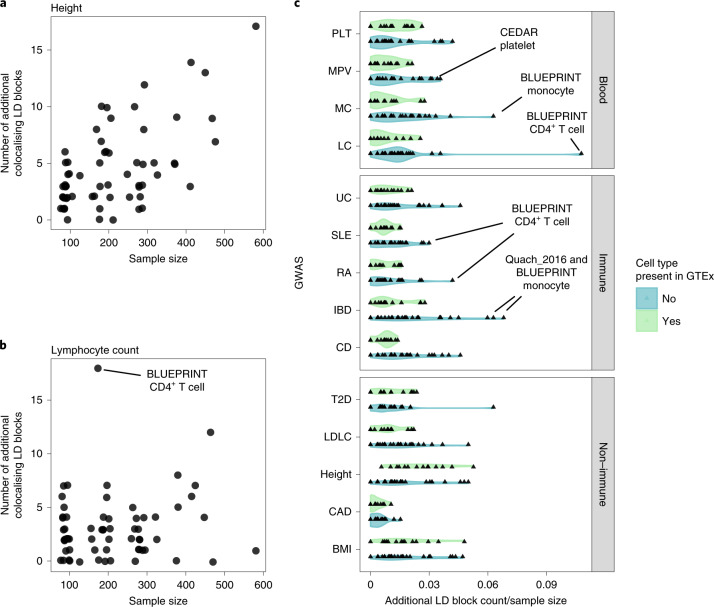
Overview of the novel GWAS co-localizations detected in the eQTL Catalogue but not in any of the GTEx tissues. **a**, The number of new height GWAS loci that co-localize with eQTLs in each cell type or tissue as a function of eQTL dataset size. **b**, The number of new lymphocyte count GWAS loci that co-localize with eQTLs in each cell type or tissue as a function of eQTL dataset size. **c**, The number of new co-localizing loci detected for the 14 GWAS traits in each cell type and tissue from the eQTL Catalogue divided by the eQTL sample sizes. The eQTL Catalogue cell types and tissues were grouped according to whether they were present in GTEx (blood, LCLs, adipose, muscle, skin and brain) or not (T cells, B cells, monocytes, macrophages, neutrophils and iPSCs). GWAS traits: PLT, MPV, MC, LC, UC, SLE, RA, IBD, CD, T2D, height, CAD, BMI and LDLC. The same analysis for the other three quantification methods is presented in Extended Data Fig. 9.

However, we often detected many additional co-localizations even in those eQTL Catalogue datasets that were already captured by GTEx (for example, blood, skin, muscle, adipose and brain tissues; Fig. 2a). These additional co-localizations could be due to thresholding effects (just below or above the PP4 ≥ 0.8 threshold), increased sample sizes in the eQTL Catalogue, or biological and population differences between datasets or other technical factors. For example, we found that the number of additional co-localizations detected for height GWAS increased linearly with the eQTL sample size, with no particular dataset standing out (Fig. 5a). In contrast, for some trait and eQTL dataset pairs, we detected considerably more co-localizations than we would have expected at the given sample size. For example, we observed 18 additional co-localizations with lymphocyte count in BLUEPRINT CD4^+^ T cells (including the *RBMS1* example in Fig. 4c), which was three times more than in any other dataset of comparable sample size (Fig. 5b).

To assess whether some eQTL datasets were particularly relevant for specific GWAS traits, we assigned each dataset a ‘novelty score’ by dividing the number of additional co-localizations detected in that dataset by its sample size. For each GWAS trait, we then asked whether the novelty scores were higher for datasets from cell types and tissues missing in GTEx compared with the datasets that were already well captured by GTEx. Although there was considerable overlap between the two distributions (Fig. 5c), we detected several trait–dataset pairs where the number of new co-localizations observed was higher than expected for a given sample size. For example, we observed most additional co-localizations for monocyte and lymphocyte count in the BLUEPRINT monocyte and CD4^+^ T-cell datasets, respectively (Fig. 5c). Similarly, we observed an excess of additional co-localization with multiple immune-mediated diseases in several monocyte and T-cell datasets (Fig. 5c). These results suggest that many additional co-localizations detected in the eQTL Catalogue relative to GTEx cannot be explained by sampling or technical variation alone and are likely to reflect cell-type-specific genetic effects.

### A subset of co-localizations manifest at the transcript level

**Fig. 6:**
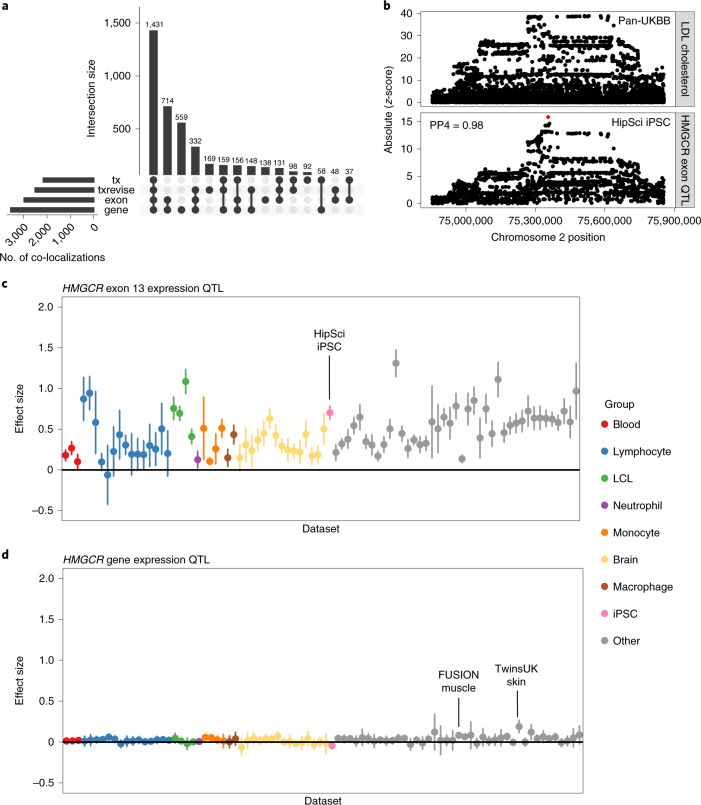
Co-localization between transcript-level QTLs and complex traits. **a**, Complex trait co-localizations (independent LD blocks) stratified by the quantification methods with which they were detected. In addition to gene-level eQTLs, we also used three transcript-level quantification methods (exon expression (exon); transcript usage (tx); and promoter, splicing and 3ʹ-end usage events (txrevise)). **b**, Regional association plot for LDL-cholesterol (top panel) and *HMGCR* exon 13 QTL in the HipSci iPSC dataset. SuSiE fine mapped the exon QTL to a single intronic variant (rs3846662, represented by the red dot), which was missing from the GWAS summary statistics. **c**, Variation in the *HMGCR* exon 13 expression QTL (rs3846662) effect sizes across eQTL Catalogue datasets. The points represent the eQTL effect size estimates from the linear model, and the error bars represent 95% confidence intervals. The HipSci iPSC dataset (*n* = 322) has been highlighted. **d**, Variation in the *HMGCR* gene expression QTL (rs3846662) effect sizes across eQTL Catalogue datasets. The points represent the eQTL effect size estimates from the linear model, and the error bars represent 95% confidence intervals. FUSION muscle (*n* = 288) and TwinsUK skin (*n* = 370) datasets have been highlighted. Sample sizes for other datasets are presented on Fig. 2a and in Supplementary Table 2.

Multiple studies have demonstrated that some co-localizations between QTLs and complex traits manifest only at the level of RNA splicing and transcript usage^36,37^. To quantify this in the eQTL Catalogue, we performed co-localization analysis across the 14 complex traits mentioned above and all QTLs detected with the 3 transcript-level quantification methods (Extended Data Fig. 1). We found that 713/4,270 (16.7%) co-localizations in independent LD blocks were detected only using 1 of the 3 transcript-level traits and not by traditional eQTLs in any of the 95 RNA-seq datasets (Fig. 6a). However, this is likely to be underestimated because transcript and gene-level QTLs could be co-localizing with independent GWAS signals within the same LD block^1^. Furthermore, our gene expression quantification was based on the total read count, which can also capture larger splicing changes, especially as the number of datasets and their sample sizes increase.

**Extended Data Fig. 10:**
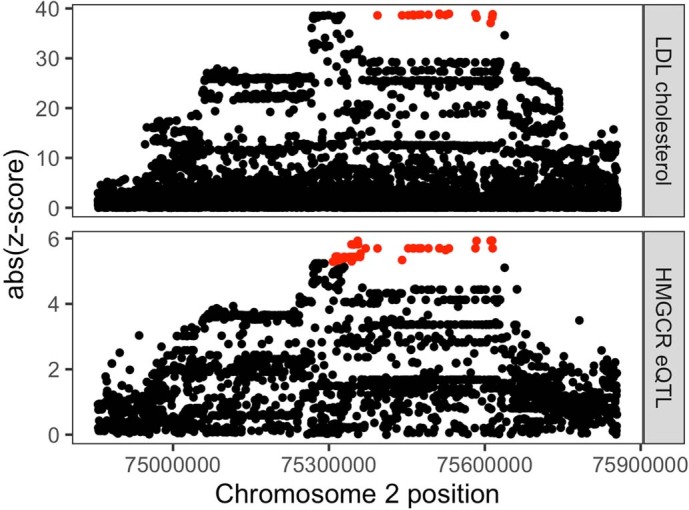
Regional association plot for LDL cholesterol (top panel) and HMGCR eQTL in the FUSION muscle dataset (bottom panel). The eQTL signal was fine mapped to 46 variants represented by red dots on both panels.

To illustrate how splicing changes can sometimes manifest as standard eQTLs, we looked at the co-localization between low-density lipoprotein (LDL)-cholesterol and an exon expression QTL for *HMGCR*. The gene product of *HMGCR* is a known target for statins, and the link between exon 13 inclusion and circulating LDL-cholesterol levels has been reported previously^37,38^. Our analysis detected co-localization (PP4 ≥ 0.8) between the expression of exon 13 of the *HMGCR* gene and LDL-cholesterol in 63/95 datasets. We saw the strongest association in the HipSci^6^ iPSC dataset, where we were able to fine map the exon QTL to a single causal variant (rs3846662, posterior probability = 1) (Fig. 6b). The same co-localization was also detected by transcript usage in 18/95 datasets and by txrevise in 29/95 datasets. Although the co-localization was also seen at the level of gene expression in the FUSION^32^ muscle dataset (PP4 = 0.99; Extended Data Fig. 10), the 95% credible set contained a total of 46 variants. Furthermore, the standardized effect size of the fine-mapped variant on exon expression (Fig. 6c) was considerably larger than on gene expression (Fig. 6d) in all datasets (Fig. 6c,d). Thus, even though some transcript-level QTLs can manifest as standard eQTLs in large datasets, having access to summary statistics from different quantification methods can inform on the identity and functional impact of the causal variant, as well as provide stronger genetic instruments for future Mendelian randomization applications.

## Discussion

We believe that the main value of the eQTL Catalogue (https://www.ebi.ac.uk/eqtl) lies in the uniformly processed gene-level and transcript-level QTL summary statistics and statistical fine-mapping results. We have thus sought to make the data as easy to use as possible. By mapping cell and tissue types to standard ontology terms, we make it easy to discover which studies contain the tissues and cell types of interest to the users. We have further re-imputed genotypes using the 1000 Genomes phase 3 reference panel for all studies using genotyping microarrays, ensuring that the same set of genetic variants is present in most studies. We have used a consistent set of molecular trait identifiers (genes, exons, transcripts, events) across all datasets, ensuring that genetic effects can be directly compared across datasets. Finally, we have released credible sets from statistical fine-mapping analysis, which can help to further characterize loci with multiple independent signals and pave the way for fine-mapping-based co-localization approaches^25^. We will progressively expand the resource to all accessible human datasets.

The relationship between gene expression similarity and eQTL sharing has been noticed before. For example, two studies conducted in stimulated monocytes and macrophages found that the number of differentially expressed genes between cell states correlates with the number of state-specific eQTLs^37,39^. This correlation raises an exciting prospect that, once a sufficient sample size has been reached in a given cell type or tissue, the discovery of new eQTL can be maximized by focusing on cell types and cell states with low gene-expression similarity to existing eQTL datasets. Of course, the definition of what is a sufficient sample size depends on how the eQTL datasets are being used. Although many cell-type- and tissue-specific *cis*-eQTLs can be detected with a sample size of a few hundred individuals, other applications such as expression-mediated heritability analysis^4^, Mendelian randomization^18^ and *trans*-eQTL analysis^2^ benefit from much larger sample sizes.

As the number of eQTL studies and their sample sizes increase, it is becoming increasingly clear that eQTL analysis is not the silver bullet for identifying causal genes underlying GWAS associations that it was once hoped to be. A number of carefully conducted studies have demonstrated that eQTL co-localization analysis often identifies multiple candidate genes, many of which are unlikely to be truly causal^40,41^. Similarly, we found that, in our analysis, 56% of LD blocks co-localized with the expression of more than one gene. The two main reasons for this are: (1) multiple independent causal variants affecting the two traits that current co-localization methods fail to properly distinguish, and (2) truly pleiotropic variants that affect multiple neighboring genes. Although improved fine mapping and co-localization methods can overcome the first limitation, true molecular pleiotropy will remain. For example, we found that 18.4% of confidently fine-mapped eQTLs (credible set size <30) were associated with the expression of two or more genes. Similarly, clustered regularly interspaced short palindromic repeats (CRISPR) perturbation experiments have shown that individual enhancers often regulate multiple neighboring genes^42^. A promising avenue to overcome this limitation are pleiotropy-robust multivariable Mendelian randomization approaches that jointly model the effects of multiple independent genetic variants across all neighboring genes to identify the most likely causal genes^43,44^, but generalizing these approaches across multiple tissues is still an open question. There is also an inherent trade-off between sensitivity and specificity. Limiting eQTL overlap analysis only to loci where the GWAS and eQTL signals are both fine mapped to a single causal variant is likely to yield high specificity^25^, but will exclude many other loci with more complex LD structure or traits that lack fine-mapping results altogether. Finally, as our *RBMS1* example highlighted, even if eQTL analysis fails to pinpoint a single causal gene, it can sometimes still reveal the most relevant cell type or context for the disease.

A limitation of our automated RNA-seq processing and eQTL mapping workflow is that we have not tailored our analyses to specific studies. For example, although the TwinsUK^33^ and HipSci^6^ studies collected samples from multiple related individuals, we used only a subset of samples (TwinsUK: 1,364 of 2,505 total; HipSci: 322 of 513 total) from unrelated individuals to avoid pseudoreplication when using linear regression. Similarly, for the six studies containing individuals from non-European and admixed populations, we jointly analyzed all samples with six genotype principal components (PCs) as co-variates. However, stratified analyses^45^ or approaches taking into account local ancestry^46,47^ might be more appropriate in this specific setting. Access to individual-level data will enable us to revisit these decisions as new analytical approaches and computational workflows become available.

A number of single-cell RNA-seq eQTL datasets have been published from differentiating iPSCs and peripheral blood cells^10,48–50^ and many others are likely to follow in the near future. These approaches are likely to revolutionize our understanding of cell-type-specific gene regulation in complex tissues and we are planning to start incorporating these datasets into the eQTL Catalogue as the raw data become available. At the same time, single-cell (sc)RNA-seq data also bring many additional challenges. To obtain the large number of cells required for eQTL mapping, many studies are relying on droplet-based scRNA-seq protocols that can only capture 5ʹ- or 3ʹ-ends of transcripts and might thus miss most genetic effects on RNA splicing. Similarly, single-cell eQTL datasets might have lower power to detect eQTLs compared with bulk, but this can be improved with proper modeling of batch effects^51^. Thus, bulk eQTL datasets are likely to remain relevant for some time as the single-cell technologies continue to improve.

To ensure that the eQTL Catalogue is a comprehensive resource that encompasses tissue and human population diversity, we encourage researchers to contribute their eQTL datasets (contact eqtlcatalogue@ebi.ac.uk). Unfortunately, we have been unable to include some existing datasets due to consent limitations or restrictions on sharing individual-level genetic data. These limitations could be overcome in the future by federated data analysis approaches, where the eQTL analysis is performed at remote sites using our analysis workflows, and only summary statistics are shared with the eQTL Catalogue. To this end, we will continue to improve the usability and portability of our data analysis workflows and will make them available via community efforts such as the nf-core^52^ repository.

## Methods

### Data access and informed consent

Gene expression and genotype data from two studies (GEUVADIS and CEDAR) were available for download without restrictions from ArrayExpress^53^. For all other datasets, we applied for access via the relevant data access committees. In our applications, we explained the project and our intent to share the association summary statistics publicly. Ethical approval for the project was obtained from the Research Ethics Committee of the University of Tartu (approval 287/T-14).

### Genotype data quality control and imputation

We aligned the strands of the genotyped variants to the 1000 Genomes phase 3 reference panel using Genotype Harmonizer^54^ v.1.4.20. We excluded genetic variants with a Hardy–Weinberg *P* value <10^−6^, missingness >0.05 and minor allele frequency (MAF) <0.01 from further analysis. We also excluded samples with >5% of their genotypes missing.

We pre-phased and imputed the genotypes to the 1000 Genomes phase 3 reference panel^30^ using Eagle v.2.4.1 (ref. ^55^) and Minimac4 (ref. ^56^) v.1.0.2. After imputation, we converted the coordinates of genetic variants from the GRCh37 reference genome to the GRCh38 using CrossMap v.0.4.1 (ref. ^57^). We used bcftools v.1.9.0 to exclude variants with MAF < 0.01 and imputation quality score *R*^2^ < 0.4 from downstream analysis. The genotype imputation and quality control steps are implemented in eQTL-Catalogue/genimpute (v.20.11.1) workflow available from GitHub (see URLs).

We used PLINK^58^ v.1.9.0 with ‘--indep-pairwise 50000 200 0.05’ to perform LD pruning of the genetic variants and LDAK^59^ v.5.0 to project new samples to the PCs of the 1000 Genomes phase 3 reference panel^30^. To assign each genotyped sample to one of four superpopulations, we calculated the Euclidean distance in the PC space from the genotyped individual to all individuals in the reference dataset. Distance from a sample to a reference superpopulation cluster is defined as a mean of distances from the sample to each reference sample from the superpopulation cluster. We explored distances between samples and reference superpopulation clusters using different numbers of PCs, and found that using three PCs worked best for inferring the superpopulation of a sample. Then, we assigned each sample to a superpopulation if the distance to the closest superpopulation cluster was at least 1.7× smaller than the second closest one (Supplementary Fig. 1a–d). We used this relatively relaxed threshold because our aim was to get an approximate estimate of the number of individuals belonging to each superpopulation. The population assignment steps are implemented in the eQTL-Catalogue/qcnorm (v.20.12.1) workflow available from GitHub (see URLs).

### Microarray data pre-processing

All five microarray studies currently included in the eQTL Catalogue (CEDAR^60^, Fairfax_2012 (ref. ^61^), Fairfax_2014 (ref. ^62^), Kasela_2017 (ref. ^63^) and Naranbhai_2015 (ref. ^64^)) used the same Illumina HumanHT-12 v.4 gene expression microarray. Batch effects, where applicable, were adjusted for with the function removeBatchEffect from the R v.3.40.6 limma package^65^. The batch adjusted log_2_(intensity values) were quantile normalized using the lumiN function from the R v.2.36.0 lumi package^66^. Only the intensities of 30,353 protein-coding probes were used.

We used Genotype harmonizer^54^ v.1.4.20 to convert the imputed genotypes into TRITYPER format. We used MixupMapper^67^ v.1.4.7 to detect sample swaps between gene expression and genotype data. We detected 155 sample swaps in the CEDAR dataset, most of which affected the neutrophil samples. We also detected one sample swap in the Naranbhai_2015 dataset.

### RNA-seq data pre-processing

The eQTL Catalogue contains RNA-seq data from the following 16 studies: ROSMAP^68^, BrainSeq^69^, TwinsUK^33^, FUSION^32^, BLUEPRINT^20,70^, Quach_2016 (ref. ^71^), Schmiedel_2018 (ref. ^21^), GENCORD^72^, GEUVADIS^73^, Alasoo_2018 (ref. ^74^), Nedelec_2016 (ref. ^75^) Lepik_2017 (ref. ^76^), HipSci^6^, van_de_Bunt_2015 (ref. ^77^) Schwartzentruber_2018 (ref. ^78^) and GTEx^1^. For each study, we downloaded the raw RNA-seq data from one of the six databases (European Genome-phenome Archive (EGA), European Nucleotide Archive (ENA), ArrayExpress, Gene Expression Omnibus (GEO), Database of Genotypes and Phenotypes (dbGaP), Synapse). If the data were already in fastq format, then we proceeded directly to quantification. If the raw data were shared in BAM or CRAM format, we used the samtools collate command^79^ to collate paired-end reads and then used samtools fastq command with ‘-F 2816 -c 6’ flags to convert the CRAM or BAM files to fastq. As samples from GEO and dbGaP were stored in Sequence Read Archive (SRA) format, we used the fastq-dump command with ‘--split-files --gzip --skip-technical --readids --dumpbase --clip’ flags to convert those to fastq. The pre-processing scripts are available from the eQTL-Catalogue/rnaseq GitHub repository (see URLs).

### RNA-seq quantification

We quantified transcription at four different levels: (1) gene expression, (2) exon expression, (3) transcript usage and (4) transcriptional event usage (Extended Data Fig. 1). Quantification was performed using a customized Nextflow^80^ workflow that we developed by adding new quantification methods to an nf-core/rnaseq pipeline^52^. Before quantification, we used Trim Galore v.0.5.0 to remove sequencing adapters from the fastq files.

For gene expression quantification, we used HISAT2 v.2.1.0 (ref. ^81^) to align reads to the GRCh38 reference genome (Homo_sapiens.GRCh38.dna.primary_assembly.fa file, downloaded from Ensembl). We counted the number of reads overlapping the genes in the GENCODE v.30 (ref. ^82^) reference transcriptome annotations with featureCounts v.1.6.4 (ref. ^83^). To quantify exon expression, we first created an exon annotation file (GFF) using GENCODE v.30 reference transcriptome annotations and dexseq_prepare_annotation.py script from the DEXSeq^84^ v.1.18.4 package. We then used the aligned RNA-seq BAM files from the gene expression quantification and featureCounts with flags ‘-p -t exonic_part -f -O’ to count the number of reads overlapping each exon.

We quantified transcript and event expression with Salmon v.0.13.1 (ref. ^85^). For transcript quantification, we used the GENCODE v.30 (GRCh38.p12) reference transcript sequences (fasta) file to build the Salmon index. For transcriptional event usage, we downloaded pre-computed txrevise^37^ alternative promoter, splicing and alternative 3ʹ-end annotations corresponding to Ensembl v.96 from Zenodo (10.5281/zenodo.3232932) in GFF format. We then used gffread^86^ v.0.9.12 to generate fasta sequences from the event annotations and built Salmon indices for each event set as we did for transcript usage. Finally, we quantified transcript and event expression using Salmon quant with ‘--seqBias --useVBOpt --gcBias --libType’ flags. All expression matrices were merged using csvtk v.0.17.0. All of these quantification methods are implemented in the eQTL-Catalogue/rnaseq workflow available from GitHub (see URLs). Our reference transcriptome annotations are available from Zenodo (10.5281/zenodo.3366280).

### RNA-seq quality control

The quality of the RNA-seq samples was assessed using the gene expression counts matrix. In all downstream analyses, we only included 35,367 protein-coding and noncoding RNA genes belonging to one of the following Ensembl gene types: lincRNA, protein_coding, IG_C_gene, IG_D_gene, IG_J_gene, IG_V_gene, TR_C_gene, TR_D_gene, TR_J_gene, TR_V_gene, 3prime_overlapping_ncrna, known_ncrna, processed_transcript, antisense, sense_intronic and sense_overlapping. For principal component analysis (PCA) and MDS analyses, we first filtered out invalid gene types (23,458) and genes on the sex chromosomes (1,247), TPM normalized^87^ the gene counts, filtered out genes having median normalized expression value <1 and log_2_ transformed the matrix. We performed PCA with the prcomp R package (center = true, scale = rue). For MDS analysis, we used the iso-MDS method from the MASS R package with *k* = 2 dimensions. As a distance metric for iso-MDS, we used 1 − Pearson’s correlation as recommended previously^88^. We plotted these two-dimensional scatter plots to visually identify outliers (Supplementary Fig. 2a,b).

Previous studies have successfully used the expression of *XIST* and Y chromosome genes to ascertain the genetic sex of RNA samples^89^. In our analysis, we extracted all protein-coding genes from the Y chromosome and the *XIST* gene (ENSG00000229807) expression values, and TPM normalized them. Then, we calculated the mean expression level of the genes on the Y chromosome. Finally, we plotted the log_2_(*XIST* expression level) (*x* axis) against the mean expression level of the genes on the Y chromosome (*y* axis). In addition to detecting samples with incorrectly labeled genetic sex, this analysis also allowed us to identify cross-contamination between samples (*XIST* and Y chromosome genes expressed simultaneously; Supplementary Fig. 2c).

Finally, we used the Match Bam to VCF (MBV) method from QTLtools^90^ which directly compares the sample genotypes in VCF format to an aligned RNA-seq BAM file. MBV can detect sample swaps, multiple samples from the same donor and cross-contamination between RNA-seq samples. In some cases, such cross-contamination was confirmed by both the sex-specific gene expression and MBV analyses (Supplementary Fig. 2d).

### RNA-seq data normalization

We excluded all samples that failed the quality control steps. We normalized the gene- and exon-level read counts using the conditional quantile normalization (cqn) R package v.1.30.0 (ref. ^91^) with gene or exon GC nucleotide content as a co-variate. We downloaded the gene GC content estimates from Ensembl biomaRt and calculated the exon-level GC content using bedtools v.2.19.0 (ref. ^92^). We also excluded lowly expressed genes, where 95% of the samples within a dataset had TPM-normalized expression <1. To calculate transcript and transcriptional event usage values, we obtained the TPM-normalized transcript (event) expression estimates from Salmon. We then divided those transcript (event) expression estimates by the total expression of all transcripts (events) from the same gene (event group). Subsequently, we used the inverse normal transformation to standardize the transcript and event usage estimates.

### Metadata harmonization

We mapped all RNA-seq and microarray samples to a minimal metadata model. This included consistent sample identifiers, information about the cell type or tissue of origin, biological context (for example, stimulation), genetic sex, experiment type (RNA-seq or microarray) and properties of the RNA-seq protocol (paired-end versus single-end; stranded versus unstranded; poly(A) selection versus total RNA). To ensure that cell type and tissue names were consistent between studies and to facilitate easier integration of additional studies, we used Zooma (https://www.ebi.ac.uk/spot/zooma) to map cell and tissue types to a controlled vocabulary of ontology terms from Uber-anatomy ontology (Uberon)^93^, Cell Ontology^94^ or Experimental Factor Ontology (EFO)^95^. We opted to use an ad-hoc controlled vocabulary to represent biological contexts because those often included terms and combinations of terms that were missing from ontologies.

### Association testing

We performed association testing separately in each dataset and used a ±1-Mb *cis* window centered around the start of each gene. First, we excluded molecular traits with fewer than five genetic variants in their *cis* window, because these were likely to reside in regions with low genotyping coverage. We also excluded molecular traits with zero variance across all samples and calculated phenotype PCs using the prcomp function from the R stats package (center = true, scale = true). We calculated genotype PCs using plink2 v.1.90b3.35. We used the first six genotype and phenotype PCs as co-variates in QTL mapping. We calculated nominal eQTL summary statistics using the GTEx v.6p of the FastQTL^96^ software (https://github.com/francois-a/fastqtl) which also estimates s.e of the effect sizes. We used the ‘--window 1000000 --nominal 1’ flags to find all associations in the 1-Mb *cis* window. For permutation analysis, we used QTLtools v.1.1 (ref. ^97^) with ‘--window 1000000 --permute 1000 --grp-best’ flags to calculate empirical *P* values based on 1,000 permutations. The ‘--grp-best’ option ensured that the permutations were performed across all molecular traits within the same ‘group’ (for example, multiple probes per gene in microarray data or multiple transcripts or exons per gene in the exon-level and transcript-level analysis) and the empirical *P* value was calculated at the group level. The steps described above are implemented in the eQTL-Catalogue/qtlmap v.21.04.1 Nextflow workflow available from GitHub (see URLs).

### Statistical fine mapping

We performed QTL fine mapping using the Sum of Single Effects Model (SuSiE)^23^ implemented in the susieR v.0.9.0R package. We converted the genotypes from VCF format to a tabix-indexed dosage matrix with bcftools v.1.10.2. We imported the genotype dosage matrix into R using the Rsamtools v.1.34.0 package. We used the same normalized molecular trait matrix used for QTL mapping and further applied a rank-based inverse normal transformation to each molecular trait, to ensure that they were normally distributed. We regressed out the first six phenotype and genotype PCs separately from the phenotype and genotype matrices. We performed fine mapping with the following parameters: *L* = 10, estimate_residual_variance = TRUE, estimate_prior_variance = TRUE, scaled_prior_variance = 0.1, compute_univariate_zscore = TRUE and min_abs_corr = 0. Finally, we extracted the 95% credible sets and the 95% posterior inclusion probabilities for each variant belonging to the credible set. The steps described above are implemented in the eQTL-Catalogue/qtlmap v.21.04.1 Nextflow workflow available from GitHub (see URLs).

### Identifying independent QTL signals

We extracted independent signals from the variants included in fine-mapped credible sets. At first, we selected credible sets with fewer than 30 variants in size and with a maximal univariate *z*-score >3. If the same credible set was associated with the expression of multiple genes (Supplementary Fig. 3), then we considered all those associations as independent signals. For every gene, we then built connected components of credible sets to represent independent signals. Two credible sets from different datasets were assigned to the same connected component if they shared at least one variant. Consequently, all variants that were part of at least one overlapping credible set were also assigned to the same connected component. To reduce the number of missing values, for each connected component, we first retained only those variants that were present in the largest number of datasets. Finally, we assigned the variant with the largest effect size (*β*) across datasets as the lead variant for each connected component. The final list contained 62,837 lead variants for 21,270 genes. We found that this approach picked slightly more lead eQTLs from datasets with smaller sample sizes, but this relationship was not strong (Supplementary Fig. 4b). We also tested an approach where the lead variant within each connected component was selected based on the smallest *P* value and found that this approach tended to favor datasets with larger sample sizes (Supplementary Fig. 4a). Reassuringly, the exact strategy for choosing lead variants did not have a strong effect on downstream eQTL sharing analysis results (Supplementary Fig. 5a–c).

Identifying independent signals for the other three quantification methods (exon expression, transcript usage and txrevise) was more challenging, because each gene often has multiple, highly correlated molecular traits (exons, transcripts, transcriptional events) that cannot be treated as independent measurements. Thus, for each gene, we first selected the smallest credible set in each dataset. If there were multiple credible sets of the same size, we selected the one containing a variant with the largest maximal posterior inclusion probability value. Then, among the selected credible sets per dataset, we randomly selected one credible set per gene across datasets. Finally, to reduce the number of missing values, for each selected credible set, we first retained only those variants that were present in the largest number of datasets and assigned the variant with the largest effect size (*β*) across datasets as the lead variant. For consistency, we also repeated the same lead variant selection process for gene expression QTLs, and again found only a weak negative relationship between dataset sample size and the number of lead variants selected from that dataset (Supplementary Fig. 4c).

### Quantifying eQTL sharing between datasets

We aggregated the eQTL data into a matrix of effect sizes, where each row represents a lead variant and each column an eQTL dataset. We noticed that this matrix contained many missing values. Although most of the missing values were caused by the gene not being expressed in a particular cell type or tissue, some of the missing values were also caused by low allele frequency or low imputation quality score. Thus, we substituted all missing values with zeros. We then calculated pairwise Spearman’s correlation between the columns of the matrix to estimate the eQTL similarity between datasets.

As an alternative to Spearman’s correlation, we used the multivariate adaptive shrinkage (Mash)^19^ model to estimate the pairwise sharing of eQTLs between datasets. The *β* values and s.e.s of lead effects were input into the Mash model as Bhat and Shat. We set missing eQTL effect sizes to 0 and s.e.s to 1. The model was fitted with *α* = 1 (exchangeable effects model). To find candidate co-variance matrices, we discovered strong effects that are significant in at least one dataset using the get_significant_results method. Then we performed PCA on identified strong effects to obtain co-variance matrices with the cov_pca function and applied extreme deconvolution to them with cov_ed. The resulting matrices were set as candidate co-variance matrices into the model fitting. We estimated pairwise eQTL sharing between datasets with the get_pairwise_sharing method by magnitude (factor of 0.5) and sign of posterior effect estimates.

### Factor analysis

We performed factor analysis using the sn-spMF model^31^. We included the 62,837 independent gene-variant pairs detected using statistical fine mapping (see above). The input files contained effect sizes and s.e.s as reciprocals of the weights of lead effects. The missing values made up ~29% of the input effect size matrix. If the effect size estimate was missing in a given cell type or tissue, then the effect size and weight were set to zero. To find hyperparameter *K* (initial number of factors), and regularization parameters *α* and *λ*, we performed a two-level grid search. In the first level, *K* was set to 20, 30, 40 and 50, and *λ* and *α* were set in a range of 1,000–2,000 with optimization of the number of iterations of 10. In the second level, we fine-tuned the parameters by narrowing the search space to those values that lead to higher sparsity of the loading and factor matrices in the first level. At the second level, we ran the parameter optimization for 20 runs and 10 iterations each. We picked the final matrix with a high cophenetic coefficient (0.89) and 21 factors.

### Co-localization

We performed co-localization analysis on QTLs in the eQTL Catalogue against GWAS summary statistics from 14 studies downloaded from the IEU OpenGWAS database in VCF format^98,99^. Our analysis included summary statistics for inflammatory bowel disease (IBD) and its two subtypes (Crohn’s disease (CD) and ulcerative colitis (UC))^100^; rheumatoid arthritis (RA)^101^, systemic lupus erythematosus (SLE)^102^, type 2 diabetes (T2D)^103^, coronary artery disease (CAD)^104^, LDL-cholesterol^105^, four blood cell type traits (lymphocyte count (LC), monocyte count (MC), platelet count (PLT), mean platelet volume (MPV))^34^ and two anthropometric traits (height, body mass index (BMI)) from the UK Biobank^105^. The variant coordinates of the GWAS summary statistics were lifted to the GRCh38 reference genome using CrossMap^57^. We used v.3.1 of the coloc R package^106^. All analysis steps are implemented in the v.21.01.1 of the eQTL Catalogue/co-localization workflow (see URLs).

We used our uniformly processed GTEx summary statistics together with all the other summary statistics from eQTL Catalogue release 3.1. For all eQTL and GWAS dataset pairs, we performed co-localization in a ±200,000 window around each of the 62,837 fine-mapped eQTL credible set lead variants (see Statistical fine mapping above). This ensured that co-localization was also performed separately for multiple independent eQTLs of the same gene and co-localization results were obtained in datasets in which no significant eQTL was detected for a particular gene. However, as we did not use masking or conditional analysis, many secondary eQTL co-localizations could still have been missed^18,107^. Inspired by the study by Barbeira et al.^3^, we summarized strong co-localizations (PP4 ≥ 0.8) at the level of approximately independent LD blocks^35^. Positions of approximately independent LD blocks were obtained from Berisa and Pickrell^35^ and converted to GRCh38 coordinates using CrossMap^57^. If the co-localization *cis* window overlapped two or more LD blocks, then the co-localizing QTL was assigned to the LD block where the QTL lead variant was located. We defined an LD block to harbor a novel co-localization signal if there was no co-localization detected within that LD block in any of the GTEx tissues. We further excluded datasets with small sample sizes (*n* < 150) due to their low power to detect co-localizations.

As transcript usage, exon expression and txrevise contained many more redundant phenotypes (for example, multiple exons of the same gene), we limited co-localization analysis for those molecular traits to the significant lead QTL variants in each dataset only (false discovery rate (FDR) < 0.01), using the same ±200,000 *cis* window as above. To make the co-localization signals comparable across quantification methods, we also performed co-localization analysis for gene expression using significant lead QTL variants as we did for the other three quantification methods. We only included QTL and complex trait pairs with strong evidence of co-localizations (PP4 ≥ 0.8) in our analysis and summarized the results at the level of independent LD blocks, as described above. The number of LD blocks for which we detected at least one co-localizing QTL with each quantification method was visualized using the upsetR v.1.4.0 R package^108^.

### URLs

Data analysis workflows:

- RNA-seq quantification: https://github.com/eQTL-Catalogue/rnaseq
- Normalization and quality control: https://github.com/eQTL-Catalogue/qcnorm
- Genotype imputation: https://github.com/eQTL-Catalogue/genimpute
- QTL analysis and fine mapping: https://github.com/eQTL-Catalogue/qtlmap
- Co-localization: https://github.com/eQTL-Catalogue/colocalisation

Example use cases:

- Accessing eQTL Catalogue summary statistics with tabix: https://github.com/eQTL-Catalogue/eQTL-Catalogue-resources/blob/master/tutorials/tabix_use_case.md
- Python example for querying the HDF5 files: https://github.com/eQTL-Catalogue/eQTL-SumStats/blob/master/querying_hdf5_basics.ipynb

### Reporting Summary

Further information on research design is available in the Nature Research Reporting Summary linked to this article.

## Online content

Any methods, additional references, Nature Research reporting summaries, extended data, supplementary information, acknowledgements, peer review information; details of author contributions and competing interests; and statements of data and code availability are available at 10.1038/s41588-021-00924-w.

## Supplementary information

Supplementary Information Supplementary Figs. 1–5 and Note.

Reporting Summary

Peer Review Information

Supplementary Tables Supplementary Tables 1–4.

## Figures and Tables

**Extended Data Fig. 9:**
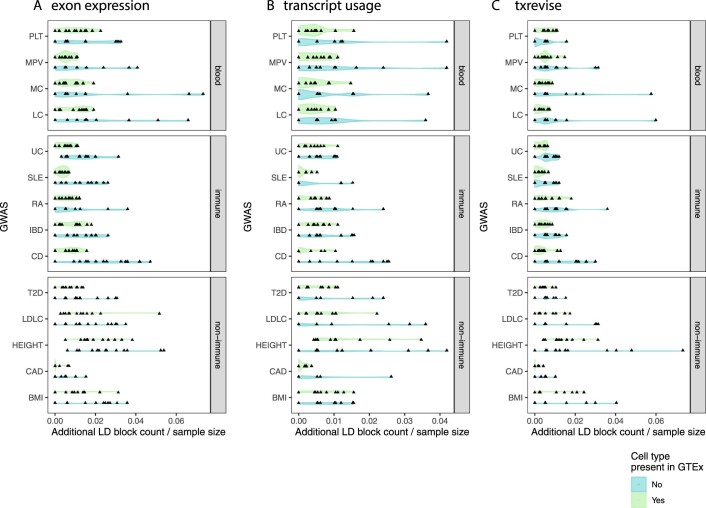
The number of additional colocalising loci detected for the 14 GWAS traits in each cell type and tissue from eQTL Catalogue divided by the eQTL sample sizes. The analysis was done independently for exon QTLs (**a**), transcript usage QTLs (**b**) and txrevise QTLs (**c**). The eQTL Catalogue cell types and tissues were grouped according to whether they were present in GTEx (blood, LCL, adipose, muscle, skin, brain) or not (T cells, B cells, monocytes, macrophages, neutrophils and iPSCs). GWAS traits: PLT - platelet count, MPV - mean platelet volume, MC - monocyte count, LC - lymphocyte count, UC - ulcerative colitis, SLE - systemic lupus erythematosus, RA - rheumatoid arthritis, IBD - inflammatory bowel disease, CD - Crohn’s disease, T2D - type 2 diabetes, height, CAD - coronary artery disease, BMI - body mass index, LDLC - LDL cholesterol.

## Data Availability

All eQTL Catalogue summary statistics are available under the Creative Commons Attribution 4.0 International License. The full association summary statistics and fine-mapped credible sets in HDF5 and TSV format can be downloaded from the eQTL Catalogue website (https://www.ebi.ac.uk/eqtl/Data_access). Slices of the TSV files can be accessed using tabix^109^ and seqminer^110^. All summary statistics are also available via the REST API (https://www.ebi.ac.uk/eqtl/api-docs). Fine-mapped credible sets can be browsed using our interactive web interface (https://elixir.ut.ee/eqtl). Our summary statistics have also been integrated into third party services such as the Open Targets Genetics Portal^111^ and FUMA^13^. The following show the accession nos. for the different data. Raw microarray gene expression data from CEDAR (E-MTAB-6667), Fairfax_2012 (E-MTAB-945), Fairfax_2014 (E-MTAB-2232) and Naranbhai_2015 (E-MTAB-3536) were downloaded from the ArrayExpress^53^. Microarray gene expression data from Kasela_2017 (GSE78840) were downloaded from the GEO. RNA-seq data from Alasoo_2018 (EGAD00001003204, PRJEB18997), GEUVADIS (E-GEUV-1), Schwartzentruber_2018 (EGAD00001003145, PRJEB18630) and HipSci (EGAD00001003529, PRJEB7388) were downloaded from the EGA and ENA. BrainSeq (syn12299750) RNA-seq data were downloaded from Synapse. Nedelec_2016 (GSE81046) RNA-seq data were downloaded from the GEO. RNA-seq and genotype data from GENCORD (EGAD00001000425, EGAD00001000428), TwinsUK (EGAD00001001086, EGAD00001001087, EGAD00001001088, EGAD00001001089), van_de_Bunt_2015 (EGAD00001001601, EGAD00001001601), Quach_2016 (EGAD00001002714, EGAD00010001131) and BLUEPRINT (EGAD00001002671, EGAD00001002674, EGAD00001002675, EGAD00001002663) were downloaded from EGA. RNA-seq and genotype data from GTEx (phs000424.v8.p2), FUSION (phs001048.v2.p1) and Schmiedel_2018 (phs001703.v1.p1) were downloaded from dbGaP. ROSMAP (syn3219045) RNA-seq and genotype data were downloaded from Synapse. HipSci, Alasoo_2018 and Schwartzentruber_2018 genotype data were downloaded from EGA and ENA (EGAD00010001147, PRJEB11752). Fairfax_2012, Fairfax_2014 and Naranbhai_2015 genotype data were downloaded from EGA (EGAD00010000144, EGAD00010000520). CEDAR (E-MTAB-6666) genotype data were downloaded from ArrayExpress. BrainSeq (phs000979.v2.p2) genotype data were downloaded from dbGaP. Lepik_2017 RNA-seq and genotype data and Kasela_2017 genotype data were obtained from the Estonian Genome Center, University of Tartu (https://genomics.ut.ee/en/access-biobank). Processed RNA-seq count matrices together with minimal metadata are available from Zenodo (10.5281/zenodo.4678936). Microarray expression matrices are available from Zenodo (10.5281/zenodo.3565554). Gene expression matrices from a subset of studies (Schwartzentruber_2018: E-ENAD-33; van_de_Bunt_2015: E-ENAD-42; HipSci: E-ENAD-35, BLUEPRINT: E-ENAD-34, Alasoo_2018: E-ENAD-41) have also been made available via the EMBL-EBI Expression Atlas^112^. We are not able to publicly share the processed genotype datasets because this is not allowed by the data-sharing conditions set by the original studies.

## Code availability

The Nextflow workflows for RNA-seq data quantification (eQTL-Catalogue/rnaseq v.20.11.1), gene expression normalization and quality control (eQTL-Catalogue/qcnorm v.20.12.1), genotype imputation (eQTL-Catalogue/genimpute v.20.11.1), QTL analysis and statistical fine mapping (eQTL-Catalogue/qtlmap v.21.04.1), and co-localization (eQTL-Catalogue/colocalisation v.21.01.1) are available from GitHub (see URLs). All code has been released under permissive MIT or Apache-2.0 license.
