# Supplementary material for: A compendium of uniformly processed human gene expression and splicing quantitative trait loci
Source: Nat Genet. 2021 Sep 6;53(9):1290–9. doi: 10.1038/s41588-021-00924-w (PMC8423625; doi:10.1038/s41588-021-00924-w)
Supplement: Supplementary file 1 — Supplementary Figs. 1–5 and Note. [file 41588_2021_924_MOESM1_ESM.pdf]

nature portfolio

https://doi.org/10.1038/s41588-021-00924-w

Supplementary information

A compendium of uniformly processed human gene expression and splicing quantitative trait loci

In the format provided by the authors and unedited

Supplementary Information

Supplementary Figures

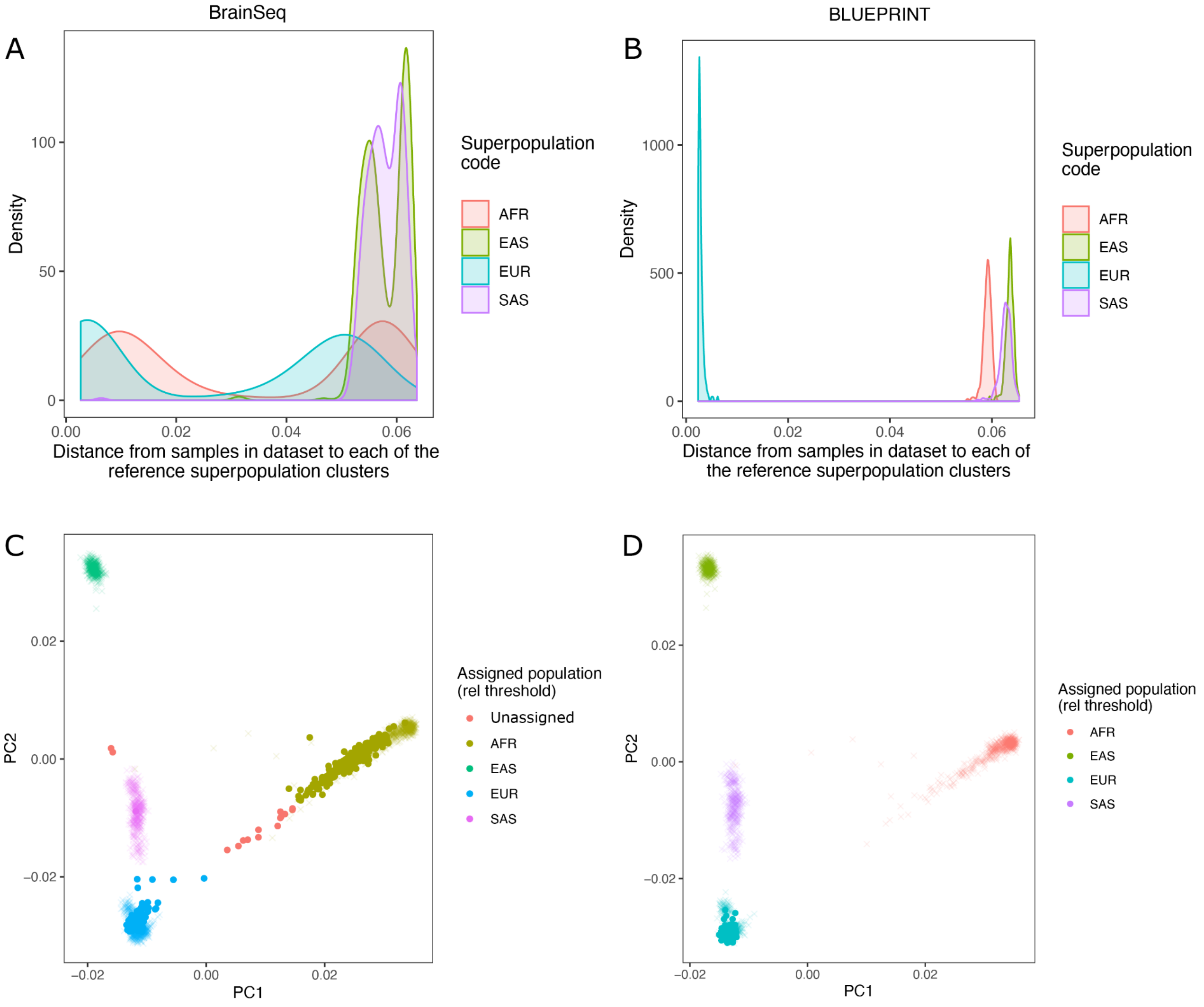

Supplementary figure 1. Assigning genotyped samples to the four 1000 Genomes superpopulations. **(A)** Density plot of distances between each sample in the BrainSeq dataset and each superpopulation cluster in the 1000 Genomes Phase 3 reference dataset. First three principal components of the genotype data are used to calculate distances. The majority of samples in the BrainSeq dataset are close to either European (EUR) or African (AFR) superpopulations. **(B)** Histogram of distances between each sample in the BLUEPRINT dataset and each superpopulation cluster in the reference dataset. All samples are close to the European (EUR) superpopulation cluster of the 1000 Genomes reference dataset. **(C)** Projection of the BrainSeq dataset to the first two principal components of the 1000 Genomes Phase 3 reference dataset. Most samples are assigned to either European or African

superpopulations. Red samples are too far from all four superpopulations and thus remain unassigned. These samples are likely to represent recent admixture. **(D)** Projection of the BLUEPRINT dataset to the first two principal components of the 1000 Genomes Phase 3 reference panel. All samples are assigned to the European superpopulation. Superpopulation codes: EUR - European, AFR - African, SAS - South Asian, EAS - East Asian.

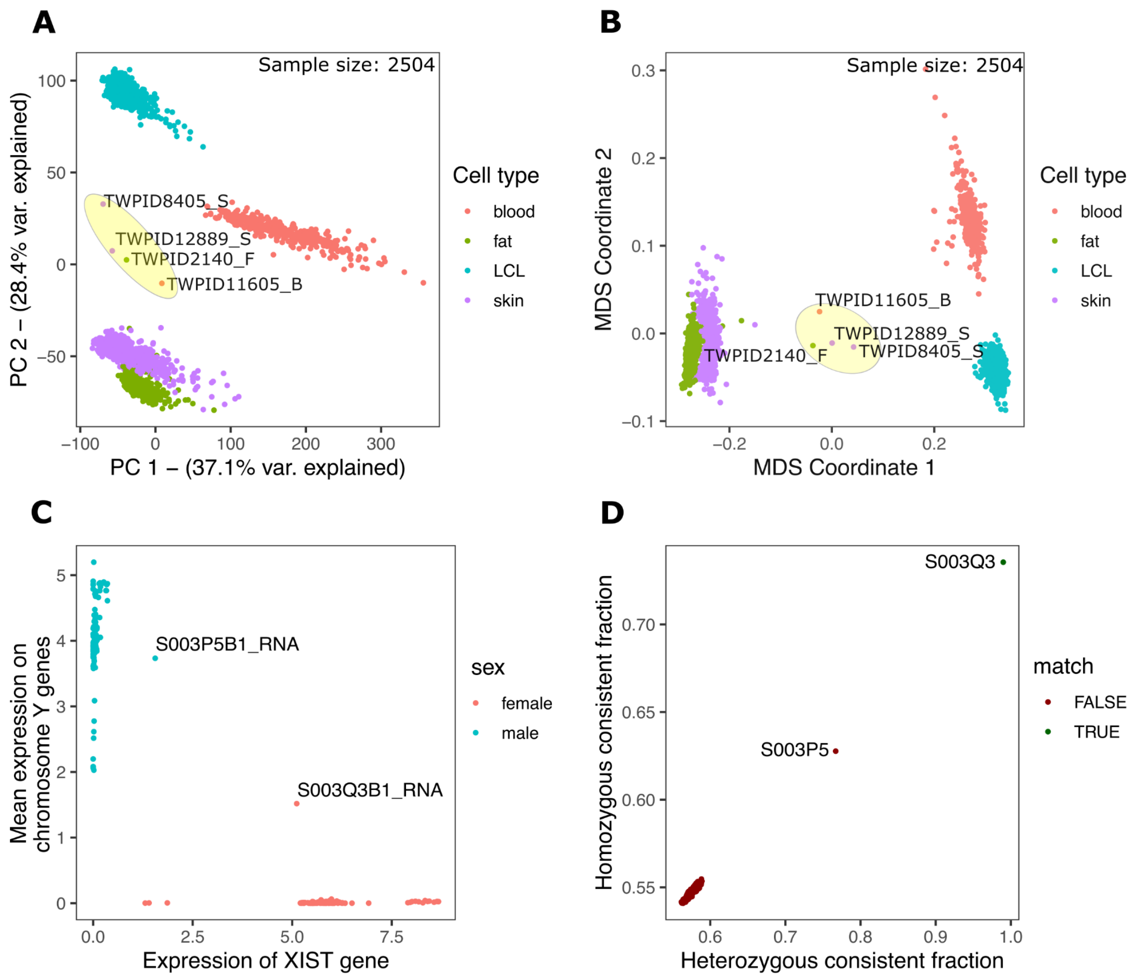

Supplementary figure 2. Overview of the Quality Control (QC) measures applied to all of the datasets in the eQTL Catalogue. QC reports for individual datasets can be found on the eQTL Catalogue website (https://www.ebi.ac.uk/eqtl/Studies/). **(A)** Principal component analysis of the TwinsUK dataset. **(B)** Multidimensional scaling analysis of the TwinsUK dataset. Four outlier samples (highlighted in yellow) from the PCA and MDS analysis were excluded from QTL mapping. (**C**) Sex-specific gene expression analysis. Expression of the female-specific *XIST* gene is plotted against the mean expression of the protein-coding genes on the Y chromosome. Samples from two donors (S003P5 (male) and S003Q3 (female)) expressed both *XIST* and genes from the Y chromosome, indicating potential cross-contamination with RNA from a sample of the opposite genetic sex. **(D)** Genetic similarity of S003Q3B1 RNA sample to all of the genotyped donors in the BLUEPRINT VCF file as calculated by the QTLtools mbv command[91]. As expected, the genotypes of the S003Q3B1 RNA sample are most similar to the genotype data from the same donor (S003Q3) and most other donors are equally dis-similar, forming a separate cluster in the bottom left corner. However, the S003Q3B1 RNA sample also displays higher-than-expected genetic similarity with genotype data from the S003P5 donor. Together with the evidence presented in panel C, this suggests that cross-contamination has occurred between the S003Q3B1 and S003P5B1 RNA samples. As a result, we decided to remove these two samples from downstream analysis.

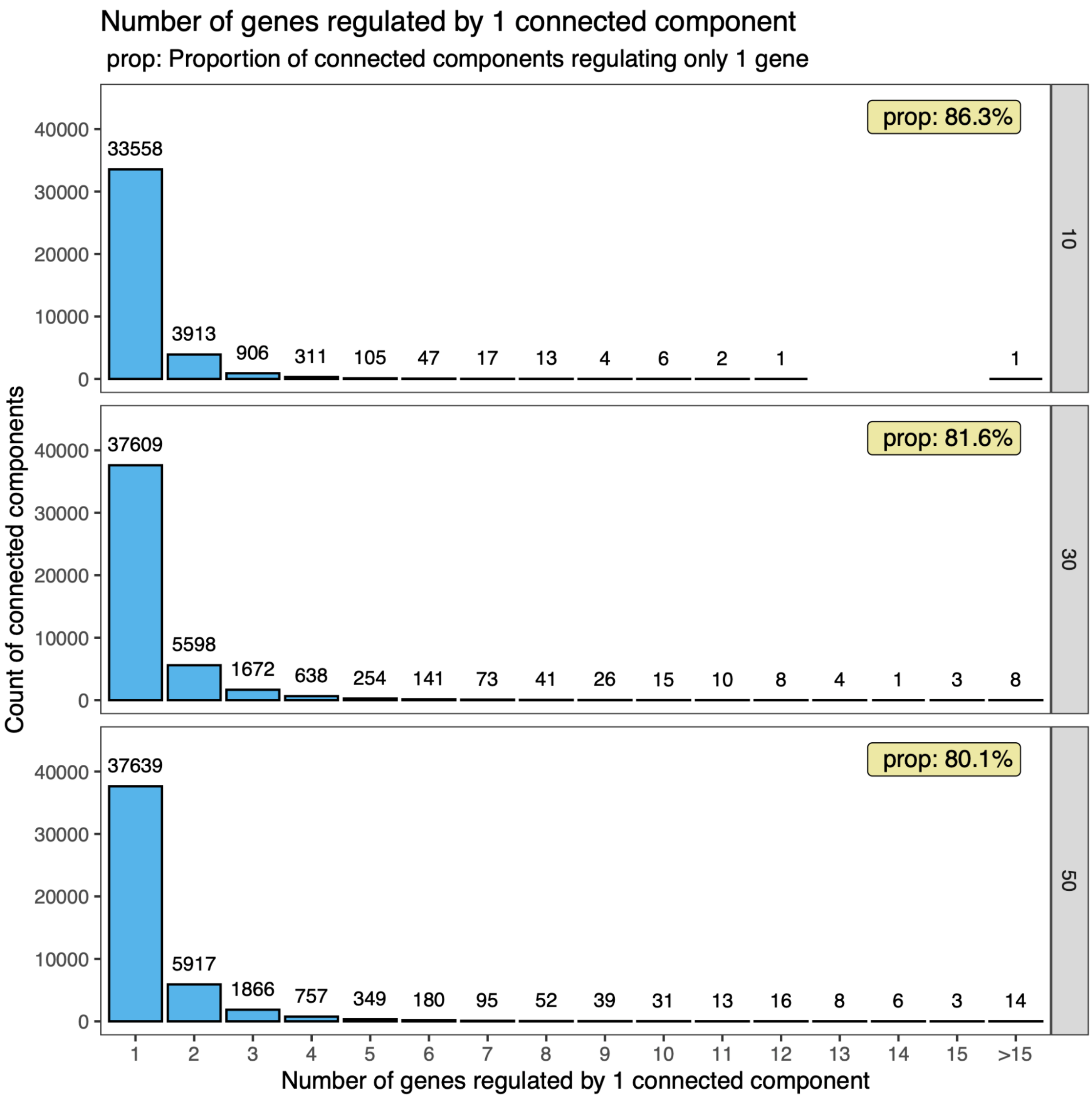

Supplementary figure 3. Estimating the number of fine mapped eQTLs that are associated with the expression of more than one gene across all 95 eQTL Catalogue datasets. We identified connected components of credible sets as described in the Methods. The three panels correspond to three maximum credible set size thresholds (n < 10, 30 or 50 variants). The eQTL sample size varied from n = 73 individuals (GTEx kidney) to n = 702 (GTEx muscle) (Supplementary Table 2).

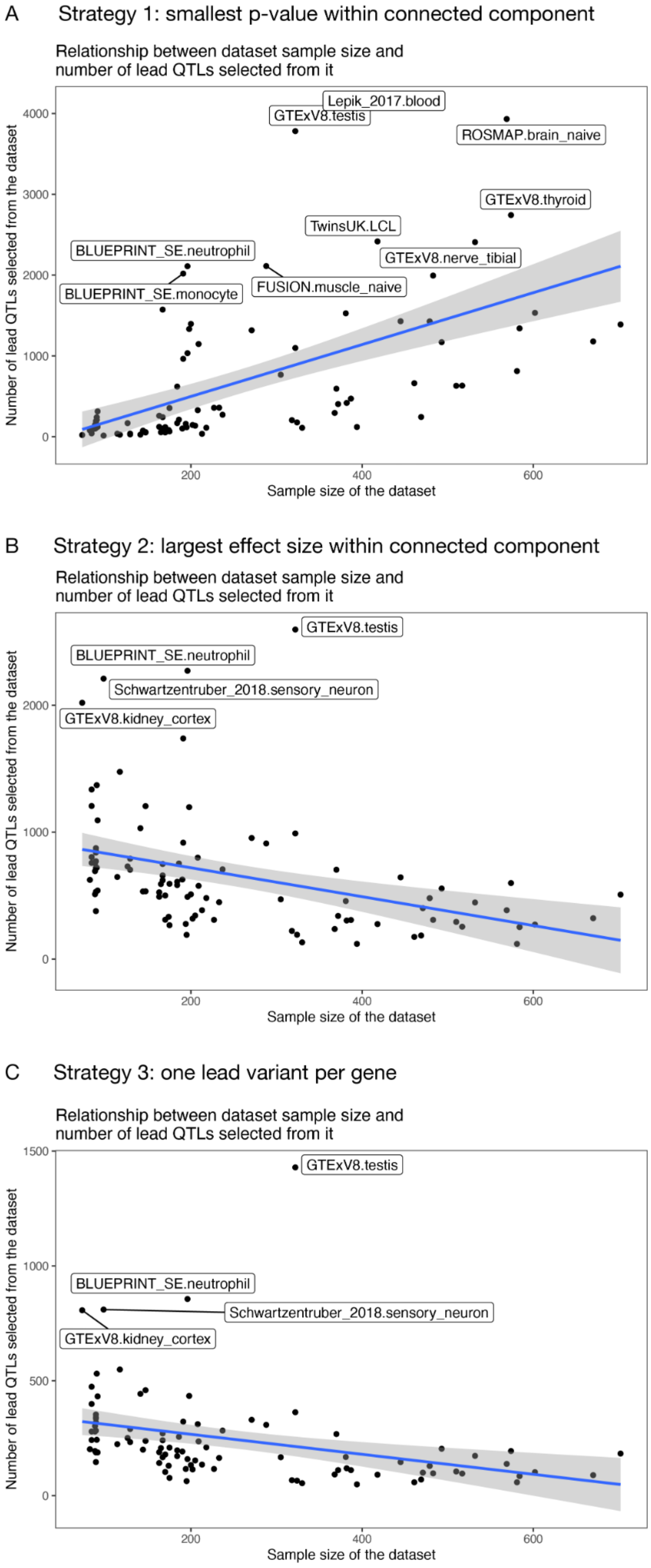

Supplementary figure 4.
Relationship between the sample size of the dataset and the number of lead eQTLs selected for eQTL sharing analysis with mash. Only credible sets with size < 30 variants were included in the analysis. The blue line represents the fitted line under linear model and the grey shadow around it represents 95% confidence interval of the fit. Number of datasets is 95 in each of the three panels. (**A**) Strategy 1 - within each connected component the variant which is present in the largest number of datasets and has the smallest p-value is selected as the lead variant. (**B**) Strategy 2 - within each connected component, the variant which is present in the largest number of datasets and has the largest effect size is chosen as the lead variant. (**C**) Strategy 3 - No connected component analysis is performed. For each gene in each dataset, we first chose the credible set with the smallest size and largest PIP value. Then we randomly selected a lead credible set per gene across datasets. Finally, we chose the variant which is present in the largest number of datasets and has the largest effect size as the lead variant of the selected credible set.

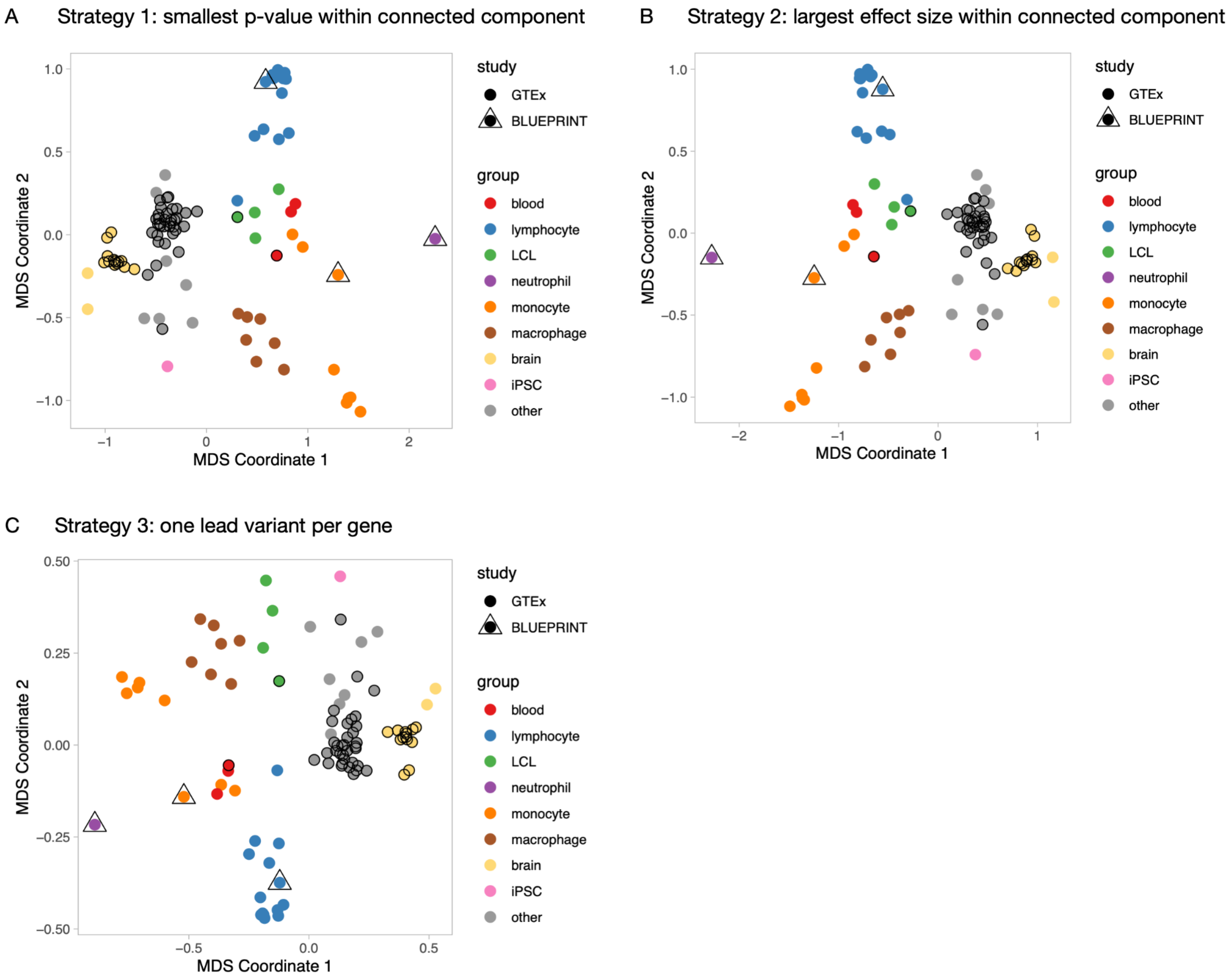

Supplementary figure 5. MDS analysis of eQTL sharing across datasets. Pairwise eQTL sharing between datasets was estimated using the Mash model. Lead variants for the Mash analysis were chosen using three alternative strategies: (**A**) Strategy 1 - within each connected component the variant which is present in the largest number of datasets and has the smallest p-value is selected as the lead variant. (**B**) Strategy 2 - within each connected component, the variant which is present in the largest number of datasets and has the largest effect size is chosen as the lead variant. (**C**) Strategy 3 - No connected component analysis is performed. For each gene in each dataset, we first chose the credible set with the smallest size and largest PIP value. Then we randomly selected a lead credible set per gene across datasets. Finally, we chose the variant which is present in the largest number of datasets and has the largest effect size as the lead variant of the selected credible set.

Supplementary Note

Funding statements for datasets in the eQTL Catalogue

BLUEPRINT. This study makes use of data generated by the Blueprint Consortium. A full list of the investigators who contributed to the generation of the data is available from www.blueprint-epigenome.eu. Funding for the project was provided by the European Union's Seventh Framework Programme (FP7/2007- 2013) under grant agreement no 282510 - BLUEPRINT.

Fairfax_2012, Fairfax_2014 and Naranbhai_2015. Funding for the project was provided by the Wellcome Trust under awards Grants 088891 [B.P.F.], 074318 [J.C.K.] and 075491/Z/04 to the core facilities at the Wellcome Trust Centre for Human Genetics, the European Research Council under the European Union's Seventh Framework Programme (FP7/2007-2013) (281824 to J.C.K.), the Medical Research Council (98082, J.C.K.) and the National Institute for Health Research (NIHR) Oxford Biomedical Research Centre.

TwinsUK. TwinsUK is funded by the Wellcome Trust, Medical Research Council, European Union, the National Institute for Health Research (NIHR)-funded BioResource, Clinical Research Facility and Biomedical Research Centre based at Guy's and St Thomas' NHS Foundation Trust in partnership with King's College London.

BrainSeq. This research was supported by the Intramural Research Program of the NIMH (NCT00001260, 900142).

Schmiedel_2018. This work was funded by the William K. Bowes Jr Foundation (P.V.) and NIH grants R24AI108564 (P.V., B.P., A.R., M.K.), S10RR027366 (BD FACSAria II), and S10OD016262 (Illumina HiSeq 2500).

ROSMAP. Study data were provided by the Rush Alzheimer's Disease Center, Rush University Medical Center, Chicago. Data collection was supported through funding by NIA grants P304G10161, R014G15819, R014G17917, R01AG30!46, R014G36836, U014G32984, U014G46152, the Illinois Department of Public Health, and the Translational Genomics Research Institute.

GENCORD. Emmanouil T Dermitzakis was supported by grants from the European Research Council (260927), Swiss National Science Foundation (31003A_130342, CRSI33_130326) Louis-Jeantet Foundation, and the Blueprint Consortium. Stylianos E Antonarakis was supported by grants from the European Research Council (249968), Swiss National Science Foundation (144082), and the Blueprint Consortium.

van_de_Bunt_2015. MvdB is supported by a Novo Nordisk postdoctoral fellowship run in partnership with the University of Oxford. ALG is a Wellcome Trust Senior Research Fellow in Basic Biomedical Science (095010/Z/10/Z). MIM is a Wellcome Trust Senior Investigator (WT098381) and a National Institute of Health Research Senior Investigator. PEM holds the Canada Research Chair in Islet Biology. This work was supported in part in Oxford, UK, by grants from the Medical Research Council (MRC; MR/L020149/1) and National Institutes of Health (NIH; R01 MH090941), and in Edmonton, Canada, by operating grants to PEM from the Canadian Institutes of Health Research (CIHR; MOP244739) and the ADI/Johnson & Johnson Diabetes Research Fund. Human islet isolations at the Alberta Diabetes Institute IsletCore

were funded by the Alberta Diabetes Foundation and the University of Alberta. The National Institute for Health Research, Oxford Biomedical Research Centre funded islet provision at the Oxford Human Islet Isolation facility. The funders had no role in study design, data collection and analysis, decision to publish, or preparation of the manuscript.

FUSION. Support for the FUSION Tissue Biopsy Study dataset was contributed by the NHGRI intramural projects ZIAHG000024 and Z1BHG000196, NIDDK grants DK062370, DK072193, and DK099240, NHGRI grant HG003079, American Diabetes Association Pathway to Stop Diabetes Grant 1-14-INI-07, and grants from the Academy of Finland.

GTEx. The Genotype-Tissue Expression (GTEx) Project was supported by the Common Fund of the Office of the Director of the National Institutes of Health (commonfund.nih.gov/GTEx). Additional funds were provided by the NCI, NHGRI, NHLBI, NIDA, NIMH, and NINDS. Donors were enrolled at Biospecimen Source Sites funded by NCI\Leidos Biomedical Research, Inc. subcontracts to the National Disease Research Interchange (10XS170), Roswell Park Cancer Institute (10XS171), and Science Care, Inc. (X10S172). The Laboratory, Data Analysis, and Coordinating Center (LDACC) was funded through a contract (HHSN268201000029C) to The Broad Institute, Inc. Biorepository operations were funded through a Leidos Biomedical Research, Inc. subcontract to Van Andel Research Institute (10ST1035). Additional data repository and project management were provided by Leidos Biomedical Research, Inc.(HHSN261200800001E). The Brain Bank was supported by supplements to the University of Miami grant DA006227. Statistical Methods development grants were made to the University of Geneva (MH090941 & MH101814), the University of Chicago (MH090951,MH090937, MH101825, & MH101820), the University of North Carolina - Chapel Hill (MH090936), North Carolina State University (MH101819), Harvard University (MH090948), Stanford University (MH101782), Washington University (MH101810), and to the University of Pennsylvania (MH101822). The datasets used for the analyses described in this manuscript were obtained from dbGaP at http://www.ncbi.nlm.nih.gov/gap through dbGaP accession number phs000424.v8.p2.
