# Supplementary material for: A compendium of uniformly processed human gene expression and splicing quantitative trait loci
Source: Nat Genet. 2021 Sep 6;53(9):1290–9. doi: 10.1038/s41588-021-00924-w (PMC8423625; doi:10.1038/s41588-021-00924-w)
Supplement: Supplementary file 2 — Reporting Summary [file 41588_2021_924_MOESM2_ESM.pdf]

Corresponding author(s): Daniel R. Zerbino, Kaur Alasoo

Last updated by author(s): Jul 7, 2021

Reporting Summary

Nature Research wishes to improve the reproducibility of the work that we publish. This form provides structure for consistency and transparency in reporting. For further information on Nature Research policies, see our Editorial Policies and the Editorial Policy Checklist.

Statistics

For all statistical analyses, confirm that the following items are present in the figure legend, table legend, main text, or Methods section.

n/a	Confirmed	
☐	☒	The exact sample size (*n*) for each experimental group/condition, given as a discrete number and unit of measurement
☐	☒	A statement on whether measurements were taken from distinct samples or whether the same sample was measured repeatedly
☐	☒	The statistical test(s) used AND whether they are one- or two-sided<br>*Only common tests should be described solely by name; describe more complex techniques in the Methods section.*
☐	☒	A description of all covariates tested
☐	☒	A description of any assumptions or corrections, such as tests of normality and adjustment for multiple comparisons
☐	☒	A full description of the statistical parameters including central tendency (e.g. means) or other basic estimates (e.g. regression coefficient) AND variation (e.g. standard deviation) or associated estimates of uncertainty (e.g. confidence intervals)
☐	☒	For null hypothesis testing, the test statistic (e.g. *F*, *t*, *r*) with confidence intervals, effect sizes, degrees of freedom and *P* value noted<br>*Give P values as exact values whenever suitable.*
☒	☐	For Bayesian analysis, information on the choice of priors and Markov chain Monte Carlo settings
☒	☐	For hierarchical and complex designs, identification of the appropriate level for tests and full reporting of outcomes
☐	☒	Estimates of effect sizes (e.g. Cohen's *d*, Pearson's *r*), indicating how they were calculated

Our web collection on statistics for biologists contains articles on many of the points above.

Software and code

Policy information about availability of computer code

Data collection	No software used
Data analysis	Workflows:<br>eQTL-Catalogue/genimpute (v20.11.1)<br>eQTL-Catalogue/rnaseq (v20.11.1)<br>eQTL-Catalogue/qcnorm (v20.12.1)<br>eQTL-Catalogue/qtlmap (v21.04.1)<br>eQTL-Catalogue/colocalisation (21.01.1)<br><br>Software tools:<br>Salmon v0.13.1<br>Samtools v1.9<br>Trim Galore v0.5.0<br>Genotype harmonizer v1.4.20<br>bcftools v1.9.0<br>plink v1.90<br>MixupMapper v1.4.7<br>Minimac4 v1.0.2<br>Eagle v2.4.1<br>bedtools v2.19.0<br>CrossMap v0.4.1<br>csvtk v0.17.0<br>featureCounts v1.6.4

Gffread v0.9.12
HISAT2 v2.1.0
QTLtools v1.1
LDAK v5.0
R 3.6.0

R packages:
DEXseq v1.18.4
upsetR v1.4.0
susieR v0.9.0
coloc v3.1
cqn v1.30.0
Rsamtools v1.34.0
limma v.3.40.6
lumi v.2.36.0

For manuscripts utilizing custom algorithms or software that are central to the research but not yet described in published literature, software must be made available to editors and reviewers. We strongly encourage code deposition in a community repository (e.g. GitHub). See the Nature Research guidelines for submitting code & software for further information.

Data

Policy information about availability of data

All manuscripts must include a data availability statement. This statement should provide the following information, where applicable:

- Accession codes, unique identifiers, or web links for publicly available datasets
- A list of figures that have associated raw data
- A description of any restrictions on data availability

All eQTL Catalogue summary statistics are available under the Creative Commons Attribution 4.0 International License. The full association summary statistics and fine mapped credible sets in HDF5 and TSV format can be downloaded from the eQTL Catalogue website (https://www.ebi.ac.uk/eqtl/Data_access/). Slices of the TSV files can be accessed using tabix and seqminer. All summary statistics are also available via the REST API (https://www.ebi.ac.uk/eqtl/api-docs/). Fine mapped credible sets can be browsed using our interactive web interface (https://elixir.ut.ee/eqtl/). Our summary statistics have also been integrated into third party services such as the Open Targets Genetics Portal and FUMA. Raw microarray gene expression data from CEDAR (E-MTAB-6667), Fairfax_2012 (E-MTAB-945), Fairfax_2014 (E-MTAB-2232) and Naranbhai_2015 (E-MTAB-3536) were downloaded from the Array Express53. Microarray gene expression data from Kasela_2017 (GSE78840) were downloaded from Gene Expression Omnibus (GEO). RNA-seq data from Alasoo_2018 (EGAD00001003204, PRJEB18997), GEUVADIS (E-GEUV-1), Schwartzentruber_2018 (EGAD0000100314, PRJEB18630) and HipSci (EGAD00001003529, PRJEB7388) were downloaded from European Genome-phenome Archive (EGA) and European Nucleotide Archive (ENA). BrainSeq (syn12299750) RNA-seq data were downloaded from Synapse. Nedelec_2016 (GSE81046) RNA-seq data were downloaded from GEO. RNA-seq and genotype data from GENCORD (EGAD00001000425, EGAD00001000428), TwinsUK (EGAD00001001086, EGAD00001001087, EGAD00001001088, EGAD00001001089), van_de_Bunt_2015 (EGAD00001001601, EGAD00001001601), Quach_2016 (EGAD00001002714, EGAD00010001131), BLUEPRINT (EGAD00001002671, EGAD00001002674, EGAD00001002675, EGAD00001002663) were downloaded from EGA. RNA-seq and genotype data from GTEx (phs000424.v8.p2), FUSION (phs001048.v2.p1) and Schmiedel_2018 (phs001703.v1.p1) were downloaded from Database of Genotypes and Phenotypes (dbGaP). ROSMAP (syn3219045) RNA-seq and genotype data were downloaded from Synapse. HipSci, Alasoo_2018 and Schwartzentruber_2018 genotype data were downloaded from EGA and ENA (EGAD00010001147, PRJEB11752). Fairfax_2012, Fairfax_2014 and Naranbhai_2015 genotype data were downloaded from EGA (EGAD00010000144, EGAD00010000520). CEDAR (E-MTAB-6666) genotype data were downloaded from ArrayExpress. BrainSeq (phs000979.v2.p2) genotype data were downloaded from dbGaP. Lepik_2017 RNA-seq and genotype data from and Kasela_2017 genotype data were obtained from the Estonian Genome Center, University of Tartu (https://genomics.ut.ee/en/access-biobank). Processed RNA-seq count matrices together with minimal metadata are available from Zenodo (https://doi.org/10.5281/zenodo.4678936). Microarray expression matrices are available from Zenodo (https://doi.org/10.5281/zenodo.3565554). Gene expression matrices from a subset of studies (Schwartzentruber_2018: E-ENAD-33; van_de_Bunt_2015: E-ENAD-42; HipSci: E-ENAD-35, BLUEPRINT: E-ENAD-34, Alasoo_2018: E-ENAD-41) have also been made available via the EMBL-EBI Expression Atlas. We are not able to publicly share the processed genotype datasets, because this is not allowed by the data sharing conditions set by the original studies.

Field-specific reporting

Please select the one below that is the best fit for your research. If you are not sure, read the appropriate sections before making your selection.

☒ Life sciences ☐ Behavioural & social sciences ☐ Ecological, evolutionary & environmental sciences

For a reference copy of the document with all sections, see nature.com/documents/nr-reporting-summary-flat.pdf

Life sciences study design

All studies must disclose on these points even when the disclosure is negative.

Sample size

The 21 datasets included in the analysis were chosen to cover as wide range of tissues, cell types and conditions as possible. They also covered the majority of eQTL datasets accessible at the beginning of the study.

Data exclusions

A small proportion of samples failing genotype or gene expression quality metrics were excluded. Summary of excluded samples is provided in Supplementary Table 4.

Replication

Our analysis included seven matching cell types and tissues (skin, adipose, LCL, blood, fibroblast, muscle, brain (DLPFC)) that were profiled in two or more studies. For all these cell types and tissues, we observed high concordance in eQTL effect sizes between studies (Figure 3C), suggesting that eQTLs replicate well between studies.

Randomization	No primary data were collected in this study.
Blinding	No primary data were collected in this study.

Reporting for specific materials, systems and methods

We require information from authors about some types of materials, experimental systems and methods used in many studies. Here, indicate whether each material, system or method listed is relevant to your study. If you are not sure if a list item applies to your research, read the appropriate section before selecting a response.

Materials & experimental systems

n/a	Involved in the study
☒	☐ Antibodies
☒	☐ Eukaryotic cell lines
☒	☐ Palaeontology and archaeology
☒	☐ Animals and other organisms
☐	☒ Human research participants
☒	☐ Clinical data
☒	☐ Dual use research of concern

Methods

n/a	Involved in the study
☒	☐ ChIP-seq
☒	☐ Flow cytometry
☒	☐ MRI-based neuroimaging

Human research participants

Policy information about studies involving human research participants

Population characteristics	No primary data were collected in this study.
Recruitment	No primary data were collected in this study.
Ethics oversight	For all controlled access datasets, we applied for access via the relevant Data Access Committees. In our applications, we explained the project and our intent to share the association summary statistics publicly. Ethical approval for the project was obtained from the Research Ethics Committee of the University of Tartu (approval 287/T-14).

Note that full information on the approval of the study protocol must also be provided in the manuscript.
