# Supplementary material for: A compendium of uniformly processed human gene expression and splicing quantitative trait loci
Source: Nat Genet. 2021 Sep 6;53(9):1290–9. doi: 10.1038/s41588-021-00924-w (PMC8423625; doi:10.1038/s41588-021-00924-w)
Supplement: Supplementary file 3 — Peer Review Information [file 41588_2021_924_MOESM3_ESM.pdf]

naturerearch

Peer Review Information

Journal: Nature Genetics
Manuscript Title: A compendium of uniformly processed human gene expression and splicing quantitative trait loci
Corresponding author name(s): Dr. Kaur Alasoo, Dr. Daniel Zerbino

Reviewer Comments & Decisions:

Decision Letter, initial version:

12th Feb 2021

Dear Dr Alasoo,

First of all, please accept my apologies for the delay in returning this decision to you.

Your Article, "eQTL Catalogue: a compendium of uniformly processed human gene expression and splicing QTLs" has now been seen by 3 referees. You will see from their comments below that while they find your work of interest, some important points are raised. We are interested in the possibility of publishing your study in Nature Genetics, but would like to consider your response to these concerns in the form of a revised manuscript before we make a final decision on publication.

To guide the scope of the revisions, the editors discuss the referee reports in detail within the team, including with the chief editor, with a view to identifying key priorities that should be addressed in revision and sometimes overruling referee requests that are deemed beyond the scope of the current study. In this case, we noted that all the reviewers were supportive of the work and have made suggestions to improve the accessibility and the overall value afforded by the resource. While Reviewers #1 and #2 have made suggestions that can be largely addressed textually, Reviewer #3 has requested some further analyses.

We invite you to revise your manuscript taking into account all reviewer and editor comments. Please highlight all changes in the manuscript text file. At this stage we will need you to upload a copy of the manuscript in MS Word .docx or similar editable format.

We are committed to providing a fair and constructive peer-review process. Do not hesitate to contact us if there are specific requests from the reviewers that you believe are technically impossible or unlikely to yield a meaningful outcome.

When revising your manuscript:

*1) Include a “Response to referees” document detailing, point-by-point, how you addressed each referee comment. If no action was taken to address a point, you must provide a compelling argument. This response will be sent back to the referees along with the revised manuscript.

*2) If you have not done so already please begin to revise your manuscript so that it conforms to our Article format instructions, available
here.
Refer also to any guidelines provided in this letter.

*3) Include a revised version of any required Reporting Summary:
https://www.nature.com/documents/nr-reporting-summary.pdf
It will be available to referees (and, potentially, statisticians) to aid in their evaluation if the manuscript goes back for peer review.
A revised checklist is essential for re-review of the paper.

Please be aware of our guidelines on digital image standards.

Please use the link below to submit your revised manuscript and related files:

[REDACTED]

Note: This URL links to your confidential home page and associated information about manuscripts you may have submitted, or that you are reviewing for us. If you wish to forward this email to co-authors, please delete the link to your homepage.

We hope to receive your revised manuscript within four to eight weeks. If you cannot send it within this time, please let us know.

Please do not hesitate to contact me if you have any questions or would like to discuss these revisions further.

Nature Genetics is committed to improving transparency in authorship. As part of our efforts in this direction, we are now requesting that all authors identified as ‘corresponding author’ on published papers create and link their Open Researcher and Contributor Identifier (ORCID) with their account on the Manuscript Tracking System (MTS), prior to acceptance. ORCID helps the scientific community achieve unambiguous attribution of all scholarly contributions. You can create and link your ORCID from the home page of the MTS by clicking on ‘Modify my Springer Nature account’. For more information please visit please visit www.springernature.com/orcid.

We look forward to seeing the revised manuscript and thank you for the opportunity to review your work.

Sincerely,

Safia Danovi
Editor
Nature Genetics

Referee expertise:

Referee #1: genetic variation and transcriptome

Referee #2: statgen and eQTL analysis

Referee #3: eQTL mapping

Reviewers' Comments:

Reviewer #1:
Remarks to the Author:
Kerimov et al. introduce eQTL catalog that provides multiple types of genetic regulatory associations for the research community, based on uniform processing of a large number of eQTL data sets. This is a very valuable resource that is already being used widely, and I believe it will become The Repository for eQTL (and sQTL etc) data and an essential resource for the genetics and genomics community.

This manuscript describes eQTL Catalog, and presents analyses of the properties and use cases of the data and demonstrate its value especially in terms of uniform effect size estimation and better fine-mapping. The biological discoveries are rather modest, but the analyses are diverse, well done, and follow all the latest standards of QTL mapping and downstream analyses. The manuscript is well written and careful work, and the data are presented very clearly. I have only a few technical comments:

- Effect size sharing analysis: "We limited our analysis to 54,733 fine mapped eQTLs (see Methods) and defined two eQTLs to be shared between a pair datasets if they had the same sign and their effect sizes did not differ more than two-fold." Wouldn't these results bias the results?
- The notion that gene expression and eQTL effect size sharing clusters very similarly could have a reference to GTEx v8 main paper (2020) that showed and quantified this pattern as well.
- It appears that the matrix factorization model was applies to only a subset of the data sets (Fig 3D). Why?
- The unit of Figure 3D color scale is not explained.
- It could be helpful to include in Fig 5 legend the definition of "novel colocalization" (i.e. not detected in GTEx) to make it clearer for a casual reader.

Reviewer #2:
Remarks to the Author:

The authors describe the identification of cis-eQTLs for a wide variety of tissues and cell-types. They use a highly consistent approach to harmonise these datasets, and therefore are able to uncover sets of robust cis-eQTLs that affect whole gene and transcript expression levels.

I am impressed by the rigour that has been applied to arrive at these results, and applaud the accompanying website. I carefully studied the materials and methods and have no comments on the procedure that has been employed to identify these cis-eQTLs. I also believe these cis-eQTLs are highly relevant for the field, particularly since in the past sometimes incorrect procedures have been employed on these dataset: these methods sometimes did not property accounted for multiple testing, resulting in a large number of reported, but false-positive eQTLs.

I do have one comment: The manuscript is a little brief on the biological interpretation or application: I would imagine that its purpose is predominantly to link associated loci for complex diseases to individual genes inside these loci. The authors use colocalization and Mendelian randomisation to do this, but I would like to see more insights in when this works, and when it doesn't:

My group has been applying coloc and MR, and we have observed that such procedures do result in the identification of positional candidate genes, but also that, based on a comparison with different gene prioritisation approaches, these candidate genes are very often not the most likely candidates. This is also something that others have recently observed (https://pubmed.ncbi.nlm.nih.gov/30239796/), and I believe the authors are uniquely positioned to report on this in the manuscript: I would love to see an analysis where the authors attempt to quantify to what extent this resource enables prioritization of the most likely positional candidate gene. Ideally, the authors might want to give recommendations on how to apply this resource, and discuss potential reasons why it remains a possibility that if for a given locus a GWAS signal and a tissue specific cis-eQTL signal are showing both colocalisation and encouraging Mendelian randomisation result that it still remains possible that this gene is in fact not the causal gene inside the locus.

It would additionally be helpful if the authors can speculate on the utility of single-cell eQTL datasets: the authors have studied several cell-type specific, but bulk RNA-seq datasets. I wonder whether single-cell RNA-seq eQTL studies might miss out on transcript eQTLs, that with bulk RNA-seq can be detected? And vice versa: what kind of biological principles can only be detected when using single-cell eQTL data instead of using bulk data instead? It might therefore be worthwhile to hear more about the pros and cons of bulk and single-cell eQTL studies.

Reviewer #3:
Remarks to the Author:
In this study the authors uniformly re-computed QTLs from 21 QTL studies, and demonstrated that cis-eQTLs are highly reproducible between studies from the same cell or tissue type. Much of the analysis resembles that of the GTEx eQTL papers, but the added value of this paper is that the observation that cell type and tissue type, not study identity or technical factors, are the dominant axis of variation in eQTL effects within this multi-study data set. I applaud the authors for their large effort of uniformly processing 21 eQTL studies, which allowed them to address this question effectively. Because this is one of the major claims of this paper, we do think that a bit more thorough analysis is needed. In particular, the overall sharing of eQTLs across tissues should be modeled

explicitly and excess enrichment of overlap across studies within tissues should be clearly demonstrated. Such enrichment can be seen in the figure, but it is not explicitly modeled and tested.

Additional comments:

The authors show eQTLs are highly reproducible between studies from the same cell or tissue type (Figure 2B, 2C). Does this hold true for the three other molecular trait (transcript usage, exon expression, txevise events) QTLs?

It was undoubtedly a large effort to uniformly process genotype, expression, and covariate data for each of the 21 studies. The impact of this study could be broadened if this intermediate data, not just summary statistics and links to the raw data from the original studies, could be made accessible.

Much of the analysis presented in the manuscript relies on the selection of the 54,733 fine-mapped eQTLs to be a representative eQTL signal. The methods section entitled "Identifying independent signals based on fine mapping" did not adequately describe how those 54,733 variants were selected. I know that SuSIE is designed to identify fine-mapped credible sets in each study, independently. How were these credible-sets merged across studies? This needs to be clarified. Furthermore, once the credible sets are merged, it is necessary to select a representative, top variant. The author's choice of selecting the variant with smallest p-value across all eQTL datasets will certainly bias variant selection in favor of higher-powered data sets. It would be useful to see that the key results (figure 2B for example) are robust to this choice.

The legend for Figure 4D does not specify what study is being colocalized with each of the eQTL Catalogue datasets. I assume it is colocalization with the GWAS hit for lymphocyte count, but this should be clarified for the reader.

Figure 5C provides compelling evidence for the importance of assaying eQTLs in diverse cell types and contexts. Does this trend hold true for the three other molecular trait eQTLs?

In line 153, the mash model is introduced as "multiple adaptive shrinkage". It is actually called "multivariate adaptive shrinkage".

Author Rebuttal to Initial comments

We have now made the following major changes to the manuscript:

1. We now use our own re-processed version of GTEx v8 summary statistics across the manuscript. As a result, the number of distinct RNA-seq samples used in the analysis increased from 17,210 to 23,839 and the number of unique donors increased from 5,714 to 6,045. Consequently, the term 'GTEx' now consistently refers to GTEx version 8 throughout the manuscript. For consistency, we have also repeated the colocalisation analysis using our re-processed GTEx summary statistics rather than the official GTEx v8 summary statistics used in the previous version of the manuscript.
2. A small bug in the release 3 of the eQTL Catalogue summary statistics caused us to underestimate the number of novel colocalisation detected in the eQTL Catalogue by approximately 50% (https://www.ebi.ac.uk/eqtl/Release_notes/). This bug was fixed in release 3.1 and the analysis in the revised version of the manuscript uses the fixed summary statistics. We now find that ~20% of the colocalisation that we detect in the eQTL Catalogue are missed when relying on GTEx data alone.
3. We have now performed several additional analyses to demonstrate that our eQTL sharing results are robust to the way in which the eQTL lead variants are selected. We also demonstrate that the main QTL sharing results generalise to the other three quantification methods.

Responses to the specific concerns raised by the reviewers can be found below.

Reviewer #1 :

Remarks to the Author:

Kerimov et al. introduce eQTL catalog that provides multiple types of genetic regulatory associations for the research community, based on uniform processing of a large number of eQTL data sets. This is a very valuable resource that is already being used widely, and I believe it will become The Repository for eQTL (and sQTL etc) data and an essential resource for the genetics and genomics community.

This manuscript describes eQTL Catalog, and presents analyses of the properties and use cases of the data and demonstrate its value especially in terms of uniform effect size estimation and better fine-mapping. The biological discoveries are rather modest, but the analyses are diverse, well done, and follow all the latest standards of QTL mapping and downstream analyses. The manuscript is well written and careful work, and the data are presented very clearly. I have only a few technical comments:

1. Effect size sharing analysis: “We limited our analysis to 54,733 fine mapped eQTLs (see Methods) and defined two eQTLs to be shared between a pair datasets if they had the same

sign and their effect sizes did not differ more than two-fold." Wouldn't these results bias the results?

We agree that how the variants are chosen for eQTL sharing analysis with mash could, in principle, bias the results. However, choosing those variants is necessary to avoid including hundreds of thousands of correlated variants into the model. To test how sensitive our results are to the choice of the variants, we have now tested three different strategies: (1) selecting the lead variant based on minimal p-value, (2) selecting the lead variants based on maximal effect size and (3) randomly selecting one lead variant per gene from the set of all fine mapped credible sets per gene. We have have now clarified this in the methods section (page 22):

Identifying independent signals based on fine mapping. *We extracted independent signals from the variants included in fine-mapped credible sets. At first, we selected credible sets with less than 30 variants in size and with a maximal univariate z-score greater than 3. For every gene, we then built connected components of credible sets to represent independent signals. Two credible sets from different datasets were assigned to the same connected component if they shared at least one variant. Consequently, all variants that were part of at least one overlapping credible set were also assigned to the same connected component. To reduce the number of missing values, for each connected component, we first retained only those variants that were present in the largest number of datasets. Finally, we assigned the variant with the largest effect size (beta) across datasets as the lead variant for each connected component. The final list contained 62,837 lead variants for 21,270 genes. We found that this approach picked slightly more lead eQTLs from datasets with smaller sample sizes, but this relationship was not strong (Supplementary Figure 14). We also tested an approach where the lead variant within each connected component was selected based on the smallest p-value and found that this approach tended to favour datasets with larger sample size (Supplementary Figure 14). Reassuringly, the exact strategy for choosing lead variants did not have a strong effect on downstream eQTL sharing analysis results (Supplementary Figure 15).*

Identifying independent signals for the other three quantification methods (exon expression, transcript usage and txrevise) was more challenging, because each gene often has multiple highly correlated molecular traits (exons, transcripts, transcriptional events) that cannot be treated as independent measurements. Thus, for each gene, we first selected the smallest credible set in each dataset. If there were multiple credible sets of the same size we selected the one containing a variant with the largest maximal PIP value. Then, among the selected credible sets per dataset, we randomly selected one credible set per gene across datasets. Finally, to reduce the number of missing values, for each selected credible set, we first retained only those variants that were present in the largest number of datasets and assigned the variant with the largest effect size (beta) across datasets as the lead variant. For consistency, we also repeated the same lead variant selection process for gene expression QTLs and again found only a weak negative

relationship between dataset sample size and the number of lead variants selected from that dataset (Supplementary Figure 14).

We found that while Strategy 1 favoured datasets with larger sample size, both strategies 2 and 3 slightly favoured datasets with smaller sample size (Supplementary Figure 14). However, the eQTL sharing patterns remained broadly similar regardless of how the lead variants were chosen (Supplementary Figure 15). In the end, we decided to use Strategy 2 (maximal effect size) for quantifying eQTL sharing and Strategy 3 for quantifying cross-dataset QTL sharing for the other three quantification methods.

2. The notion that gene expression and eQTL effect size sharing clusters very similarly could have a reference to GTEx v8 main paper (2020) that showed and quantified this pattern as well.

Thank you for the suggestion. This section of the manuscript (lines 160-163) now reads:

Consistent with previous reports by GTEx, we found that the eQTL similarity between datasets closely matched their gene expression similarity (Figure 3A-B) (1). This suggests that high gene expression similarity and a high degree of eQTL sharing both reflect similarity in the underlying regulatory state of cells.

3. It appears that the matrix factorization model was applies to only a subset of the data sets (Fig 3D). Why?

Matrix factorisation was always applied to all datasets, but to improve legibility, in the previous version of the manuscript we only included those cell types and tissues in Figure 3D that had non-zero factor loadings in at least one other factor than the universal factor. We have now repeated the matrix factorisation analysis using summary statistics from GTEx v8 and updated Figure 3D (and added Supplementary Figure 7) to contain almost all of the datasets (except nine stimulated monocyte and macrophage datasets for which we did not detect a separate factor). Fitting these additional 9 datasets on the figure would have made the factor labels too small to read.

4. The unit of Figure 3D color scale is not explained.

We have now modified the legend of Figure 3D to clairy that the colour scale represents factor loadings (in arbitrary units).

5. It could be helpful to include in Fig 5 legend the definition of "novel colocalization" (i.e. not detected in GTEx) to make it clearer for a casual reader.

The first sentence of the caption for Figure 5 now reads:

Overview of the novel GWAS colocalisations detected in the eQTL Catalogue but not in any of the GTEx tissues.

Reviewer #2:

Remarks to the Author:

The authors describe the identification of cis-eQTLs for a wide variety of tissues and cell-types. They use a highly consistent approach to harmonise these datasets, and therefore are able to uncover sets of robust cis-eQTLs that affect whole gene and transcript expression levels.

I am impressed by the rigour that has been applied to arrive at these results, and applaud the accompanying website. I carefully studied the materials and methods and have no comments on the procedure that has been employed to identify these cis-eQTLs. I also believe these cis-eQTLs are highly relevant for the field, particularly since in the past sometimes incorrect procedures have been employed on these dataset: these methods sometimes did not property accounted for multiple testing, resulting in a large number of reported, but false-positive eQTLs.

1. I do have one comment: The manuscript is a little brief on the biological interpretation or application: I would imagine that its purpose is predominantly to link associated loci for complex diseases to individual genes inside these loci. The authors use colocalization and Mendelian randomisation to do this, but I would like to see more insights in when this works, and when it doesn't:

My group has been applying coloc and MR, and we have observed that such procedures do result in the identification of positional candidate genes, but also that, based on a comparison with different gene prioritisation approaches, these candidate genes are very often not the most likely candidates. This is also something that others have recently observed (https://pubmed.ncbi.nlm.nih.gov/30239796/), and I believe the authors are uniquely positioned to report on this in the manuscript: I would love to see an analysis where the authors attempt to quantify to what extent this resource enables prioritization of the most likely positional candidate gene. Ideally, the authors might want to give recommendations on how to apply this resource, and discuss potential reasons why it remains a possibility that if for a given locus a GWAS signal and a tissue specific cis-eQTL signal are showing both colocalisation and encouraging Mendelian randomisation result that it still remains possible that this gene is in fact not the causal gene inside the locus.

First, to clarify, we have only relied on colocalisation in our analysis and did not use Mendelian randomisation. Nevertheless, we completely agree that these approaches can often point to multiple genes, many of which are probably not causally involved in the GWAS trait. We now discuss this extensively in the manuscript (lines 386-408):

As the number of eQTL studies and their sample sizes increases, it is becoming increasingly clear that eQTL analysis is not the silver bullet for identifying causal genes underlying GWAS associations that it was once hoped to be. A number of carefully conducted studies have demonstrated that eQTL colocalisation analysis often identifies multiple candidate genes, many

of which are unlikely to be truly causal (41, 42). Similarly, we found that in our analysis 56% of LD blocks colocalised with the expression of more than one gene. The two main reasons for this are: 1) multiple independent causal variants affecting the two traits that current colocalisation methods fail to properly distinguish, and 2) truly pleiotropic variants that affect multiple neighbouring genes. While improved fine mapping and colocalisation methods can overcome the first limitation, true molecular pleiotropy will remain. For example, we found that 18.4% of confidently fine mapped eQTLs (credible set size < 30) were associated with the expression of two or more genes (Supplementary Figure 11). Similarly, CRISPR perturbation experiments have shown that individual enhancers often regulate multiple neighbouring genes (43). A promising avenue to overcome this limitation are pleiotropy-robust multivariable mendelian randomisation approaches that jointly model the effects of multiple independent genetic variants across all neighbouring genes to identify the most likely causal genes (44, 45), but generalising these approaches across multiple tissues is still an open question. There is also an inherent trade-off between sensitivity and specificity. Limiting eQTL overlap analysis only to loci where the GWAS and eQTL signals are both fine mapped to single causal variant is likely to yield high specificity (25), but will exclude many other loci with more complex LD structure or traits that lack fine mapping results altogether. Finally, as our RBMS1 example highlighted, even if eQTL analysis fails to pinpoint to a single causal gene, it can sometimes still reveal the most relevant cell type or context for the disease.

2. It would additionally be helpful if the authors can speculate on the utility of single-cell eQTL datasets: the authors have studied several cell-type specific, but bulk RNA-seq datasets. I wonder whether single-cell RNA-seq eQTL studies might miss out on transcript eQTLs, that with bulk RNA-seq can be detected? And vice versa: what kind of biological principles can only be detected when using single-cell eQTL data instead of using bulk data instead? It might therefore be worthwhile to hear more about the pros and cons of bulk and single-cell eQTL studies.

We agree that single-cell eQTL datasets are going to be highly relevant in the future and we have already started to integrate the first ones to the eQTL Catalogue. To reflect their importance, we have now added the following paragraph to the discussion (lines 421-431):

A number of single-cell RNA-seq eQTL datasets have been published from differentiating iPSCs and peripheral blood cells (10, 49–51) and many others are likely to follow in the near future. These approaches are likely to revolutionize our understanding of cell-type-specific gene regulation in complex tissues and we are planning to start incorporating these datasets to the eQTL Catalogue as the raw data becomes available. At the same time, scRNA-seq data also brings many additional challenges. To obtain the large number of cells required for eQTL mapping, many studies are relying on droplet-based scRNA-seq protocols that are only able to capture 5' or 3' ends of transcripts and might thus miss most genetic effects on RNA splicing. Similarly, single-cell eQTL datasets might have lower power to detect eQTLs compared to bulk, but this can be improved with proper modelling of batch effects (52). Thus, bulk eQTL datasets

are likely to remain relevant for some time as the single-cell technologies continue to improve.

Reviewer #3:

Remarks to the Author:

In this study the authors uniformly re-computed QTLs from 21 QTL studies, and demonstrated that cis-eQTLs are highly reproducible between studies from the same cell or tissue type. Much of the analysis resembles that of the GTEx eQTL papers, but the added value of this paper is that the observation that cell type and tissue type, not study identity or technical factors, are the dominant axis of variation in eQTL effects within this multi-study data set. I applaud the authors for their large effort of uniformly processing 21 eQTL studies, which allowed them to address this question effectively. Because this is one of the major claims of this paper, we do think that a bit more thorough analysis is needed.

1. In particular, the overall sharing of eQTLs across tissues should be modeled explicitly and excess enrichment of overlap across studies within tissues should be clearly demonstrated. Such enrichment can be seen in the figure, but it is not explicitly modeled and tested.

We decided to approach this by focussing on the seven cell types and tissues that had sufficiently large sample size ($n > 200$) and were profiled in two or more studies:

- LCL: TwinsUK, GENCORD, GEUVADIS, GTEx
- Adipose: FUSION, TwinsUK, GTEx
- Skin: GTEx, TwinsUK
- Blood: Lepik_2019, GTEx, TwinsUK
- Muscle: GTEx, FUSION
- Fibroblast: GTEx, GENCORD
- Brain (DLPFC): GTEx, ROSMAP, BrainSeq

For all these cell types and tissues, we obtained their pairwise eQTL sharing estimates from mash and compared their distributions in two groups: (1) same tissue, different study vs (2) different tissue, same study. We found that the eQTL sharing estimates were significantly higher in the first group compared to the second group (Wilcoxon rank sum test p-value $< 10^{-8}$) (Supplementary Figure 5A). Importantly the results did not change if we used a different strategy to define lead eQTLs variants for the mash analysis (Supplementary Figure 5B). We also repeated the same analysis for the three other quantification methods and observed the same general trend, although the differences were slightly smaller (Supplementary Figure 5C-E).

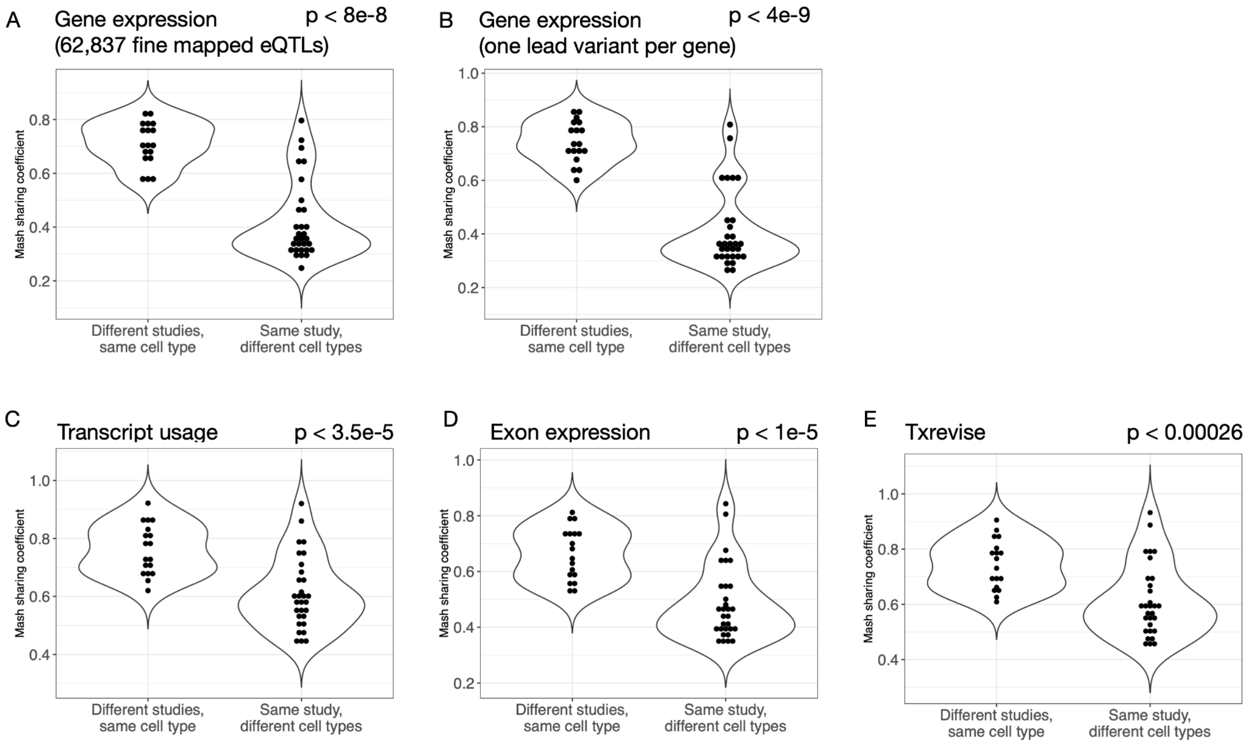

Supplementary Figure 5. Supplementary Figure 5. Distribution of pairwise mash QTL sharing estimates for seven cell types and tissues (skin, adipose, LCL, blood, fibroblast, muscle, brain (DLPFC) profiled in two or more studies (GTEx, TwinsUK, GENCORD, GEUVADIS, Lepik_2017, ROSMAP, FUSION). Each panel contrasts the QTL sharing estimates for the same cell type or tissue profiled in different studies against different cell types and tissues profiled in the same study. Analysis was performed separately for gene expression (**A-B**), transcript usage (**C**), exon expression (**D**) and txrevise (**E**) QTLs. Note that adipose, skin and muscle tissues have high eQTL sharing also within the same study. The p-values were calculated using the two-sample Wilcoxon rank sum test.

We now refer to this analysis also in the main text (lines 178-181):
To assess this formally, we focussed on a subset of tissues profiled in at least two studies. We found that the average eQTL sharing for the same tissue profiled in two different studies was significantly higher than for two different cell types or tissues profiled within the same study (Supplementary Figure 5)

Additional comments:

2. The authors show eQTLs are highly reproducible between studies from the same cell or tissue type (Figure 2B, 2C). Does this hold true for the three other molecular trait (transcript usage, exon expression, txevise events) QTLs?

We have now used mash to estimate QTL sharing between datasets also for the other three molecular traits. These results are presented in Supplementary Figure 4, which is also copied

below for convenience. Overall, we see broadly similar sharing patterns as we observed for eQTLs, but the results are noisier and there is some evidence for slightly larger batch effects (e.g. BLUEPRINT CD4+ T-cells no longer cluster as well together with other T-cell datasets and the distinction between whole blood, monocytes and LCLs is weaker).

We have added the following sentence to the main text (lines 176-178):
We also observed broadly similar patterns of sharing among the QTLs detected with the other three quantification methods (Supplementary Figure 4).

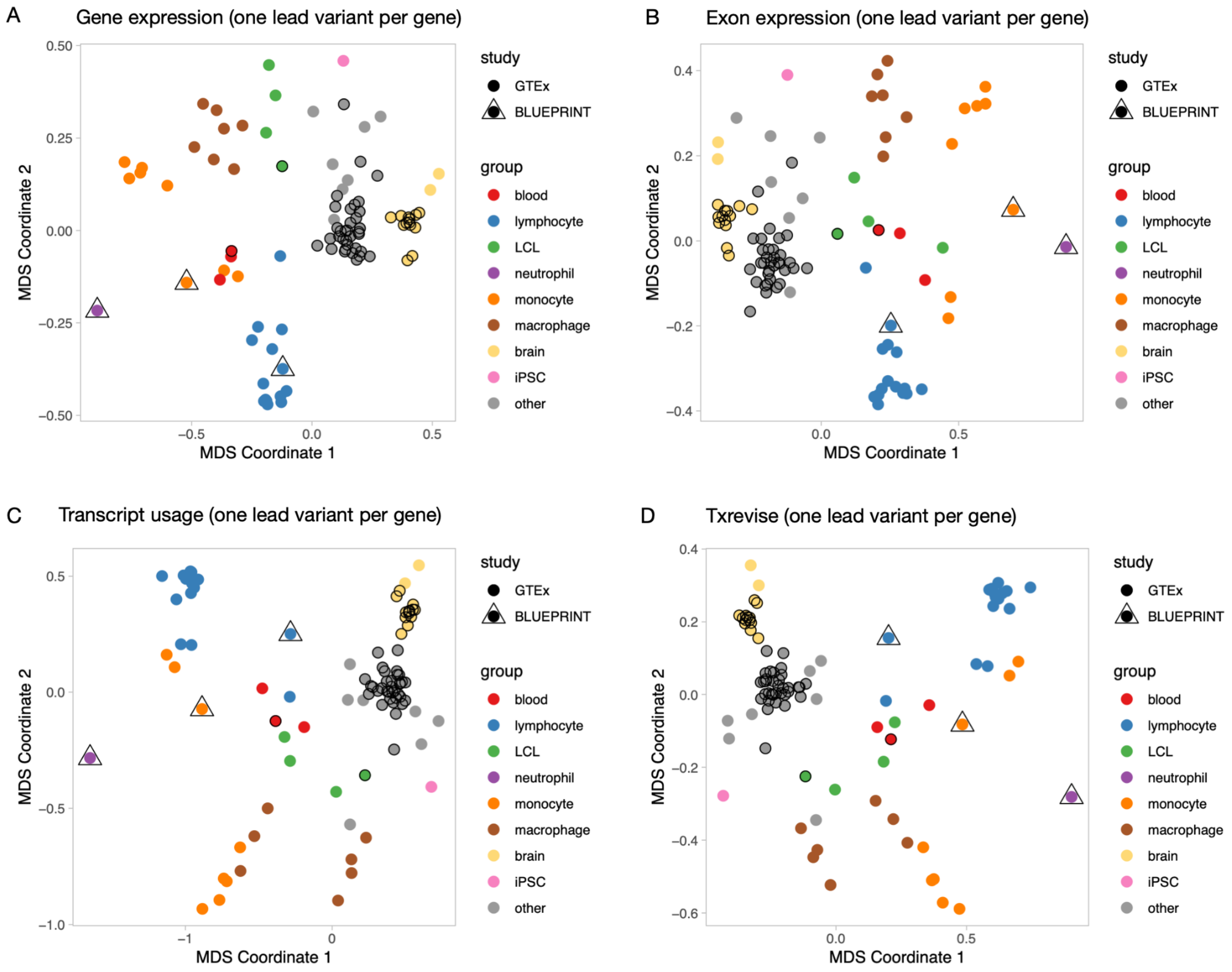

Supplementary Figure 4. MDS analysis of eQTL sharing across datasets. Pairwise eQTL sharing between datasets was estimated using the Mash model. The individual points have been coloured according to the major cell type and tissue groups. To facilitate comparison between quantification methods, and avoid redundant signals from correlated transcripts and exons, all analyses have been performed using one lead variant per gene (see Methods). The panels show pairwise eQTL sharing MDS plots for gene expression (**A**), exon expression (**B**), transcript usage (**C**) and txrevise (**D**) QTLs.

3. It was undoubtedly a large effort to uniformly process genotype, expression, and covariate data for each of the 21 studies. The impact of this study could be broadened if this intermediate data, not just summary statistics and links to the raw data from the original studies, could be made accessible.

We have now published the processed RNA-seq count matrices and microarray gene expression data together with minimal sample metadata on Zenodo and added the following clarification to the the Data availability section:

Processed RNA-seq count matrices together with minimal metadata are available from Zenodo (https://doi.org/10.5281/zenodo.4678936). Microarray expression matrices are also available from Zenodo (https://doi.org/10.5281/zenodo.3565554). We are not able to publicly share the processed genotype datasets, because this is not allowed by the data sharing conditions set by the original studies.

Unfortunately, we are unable to share the processed genotype files due to identifiable personal information contained in those and the restrictions set by the original data owners. Users who need access to the individual-level genotype data should directly contact each individual cohort. For convenience, the database accessions of the individual studies are provided on our website (https://www.ebi.ac.uk/eqtl/Studies/).

4. Much of the analysis presented in the manuscript relies on the selection of the 54,733 fine-mapped eQTLs to be a representative eQTL signal. The methods section entitled "Identifying independent signals based on fine mapping" did not adequately describe how those 54,733 variants were selected. I know that SuSIE is designed to identify fine-mapped credible sets in each study, independently. How were these credible-sets merged across studies? This needs to be clarified. Furthermore, once the credible sets are merged, it is necessary to select a representative, top variant. The author's choice of selecting the variant with smallest p-value across all eQTL datasets will certainly bias variant selection in favor of higher-powered data sets. It would be useful to see that the key results (figure 2B for example) are robust to this choice.

We agree that this part of the Methods section was unclear. We have now significantly rewritten it, adding much more detail (page 22):

Identifying independent signals based on fine mapping. *We extracted independent signals from the variants included in fine-mapped credible sets. At first, we selected credible sets with less than 30 variants in size and with a maximal univariate z-score greater than 3. For every gene, we then built connected components of credible sets to represent independent signals. Two credible sets from different datasets were assigned to the same connected component if they shared at least one variant. Consequently, all variants that were part of at least one overlapping credible set were also assigned to the same connected component. To reduce the number of missing values, for each connected component, we first retained only those variants that were*

present in the largest number of datasets. Finally, we assigned the variant with the largest effect size (beta) across datasets as the lead variant for each connected component. The final list contained 62,837 lead variants for 21,270 genes. We found that this approach picked slightly more lead eQTLs from datasets with smaller sample sizes, but this relationship was not strong (Supplementary Figure 14). We also tested an approach where the lead variant within each connected component was selected based on the smallest p-value and found that this approach tended to favour datasets with larger sample size (Supplementary Figure 14). Reassuringly, the exact strategy for choosing lead variants did not have a strong effect on downstream eQTL sharing analysis results (Supplementary Figure 15).

Identifying independent signals for the other three quantification methods (exon expression, transcript usage and txrevise) was more challenging, because each gene often has multiple highly correlated molecular traits (exons, transcripts, transcriptional events) that cannot be treated as independent measurements. Thus, for each gene, we first selected the smallest credible set in each dataset. If there were multiple credible sets of the same size then we selected the one containing a variant with the largest maximal PIP value. Then, among the selected credible sets per dataset, we randomly selected one credible set per gene across datasets. Finally, to reduce the number of missing values, for each selected credible set, we first retained only those variants that were present in the largest number of datasets and assigned the variant with the largest effect size (beta) across datasets as the lead variant. For consistency, we also repeated the same lead variant selection process for gene expression QTLs and again found only a weak negative relationship between dataset sample size and the number of lead variants selected from that dataset (Supplementary Figure 14).

Regarding robustness, we now tried three different options: (1) selecting the lead variant based on minimal p-value, (2) selecting the lead variants based on maximal effect size and (3) selecting only one lead variant per gene from a randomly selected credible set. We found that while Strategy 1 indeed favoured datasets with larger sample size, both Strategies 2 and 3 slightly favoured datasets with smaller sample size (Supplementary Figure 14). However, the eQTL sharing patterns remained broadly similar regardless of how the lead variants were chosen (Supplementary Figure 15).

The Supplementary Figures 14 and 15 are shown below for convenience:

A Strategy 1: smallest p-value within connected component

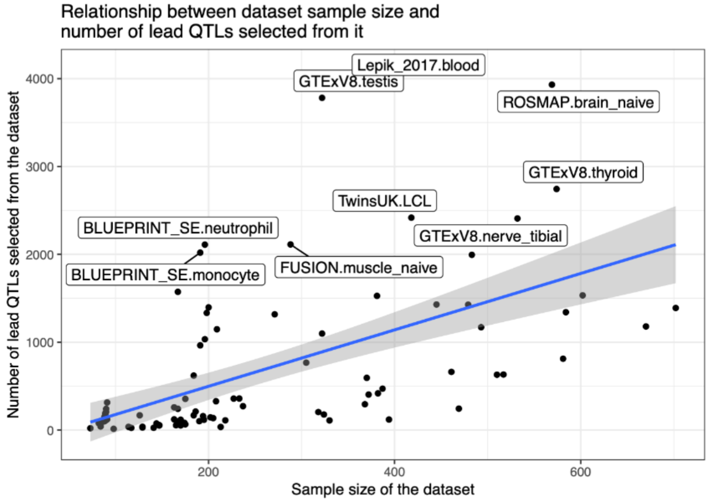

B Strategy 2: largest effect size within connected component

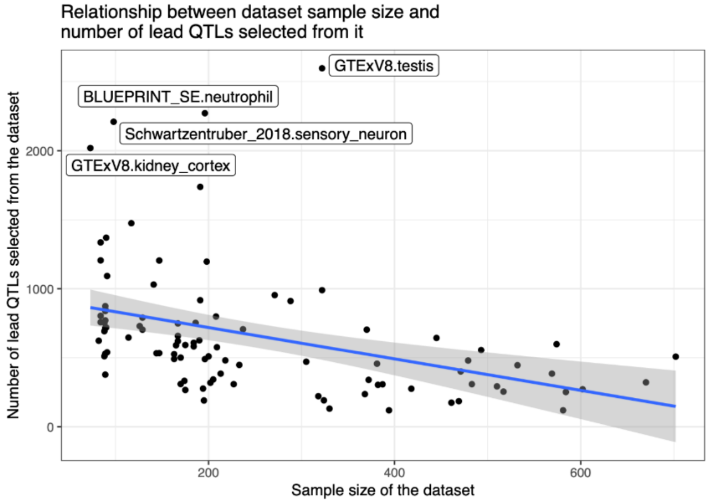

C Strategy 3: one lead variant per gene

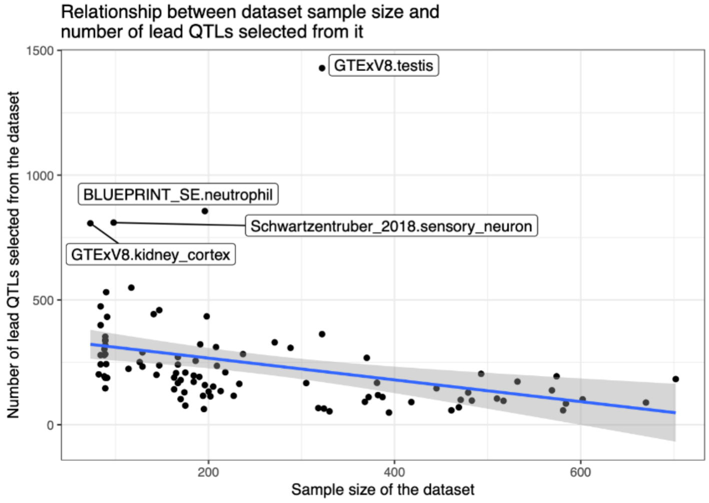

Supplementary Figure 14. Relationship between the sample size of the dataset and the number of lead eQTLs selected for eQTL sharing analysis with mash. Only credible sets with size < 30 variants were included in the analysis. (**A**) Strategy 1 - within each connected component the variant which is present in the largest number of datasets and has the smallest p-value is selected as the lead variant. (**B**) Strategy 2 - within each connected component, the variant which is present in the largest number of datasets and has the largest effect size is chosen as the lead variant. (**C**) Strategy 3 - No connected component analysis is performed. For each gene in each dataset, we first chose the credible set with the smallest size and largest PIP value. Then we randomly selected a lead credible set per gene across datasets. Finally, we chose the variant which is present in the largest number of datasets and has the largest effect size as the lead variant of the selected credible set.

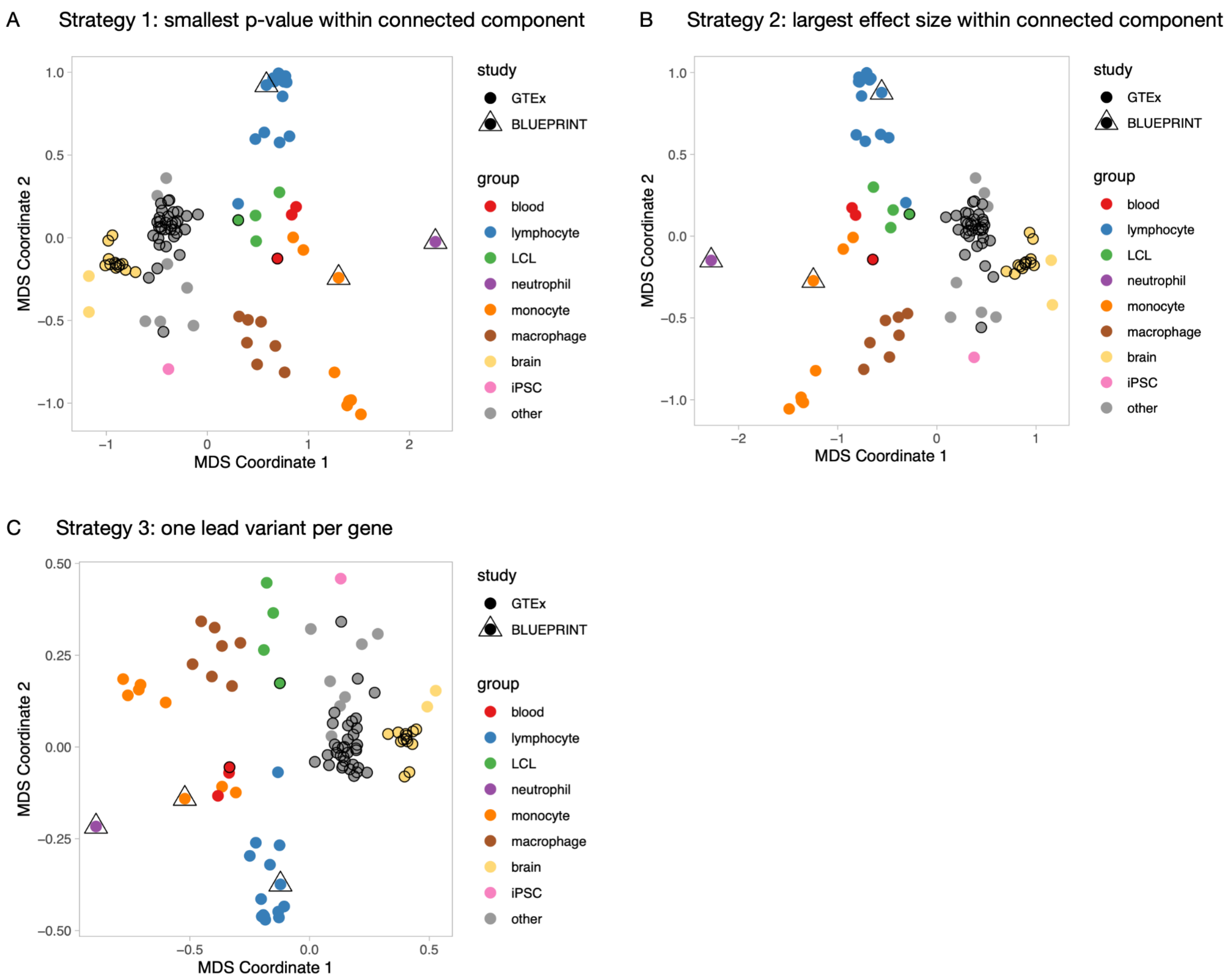

Supplementary Figure 15. MDS analysis of eQTL sharing across datasets. Pairwise eQTL sharing between datasets was estimated using the Mash model. Lead variants for the Mash analysis were chosen using three alternative strategies: (**A**) Strategy 1 - within each connected component the variant which is present in the largest number of datasets and has the smallest p-value is selected as the lead variant. (**B**) Strategy 2 - within each connected component, the variant which is present in the largest number of datasets and has the largest effect size is chosen as the lead variant. (**C**) Strategy 3 - No connected component analysis is performed. For each gene in each dataset, we first chose the credible set with the smallest size and largest PIP value. Then we randomly selected a lead credible set per gene across datasets. Finally, we chose the variant which is present in the largest number of datasets and has the largest effect size as the lead variant of the selected credible set.

5. The legend for Figure 4D does not specify what study is being colocalized with each of the eQTL Catalogue datasets. I assume it is colocalization with the GWAS hit for lymphocyte count, but this should be clarified for the reader.

The caption for Figure 4D now reads:

Colocalisation posterior probabilities between lymphocyte count and RBMS1 expression in the region surrounding the eQTL lead variant (rs6753933) across all eQTL Catalogue datasets. PP4 represents a shared causal variant while PP3 represents two distinct causal variants.

6. Figure 5C provides compelling evidence for the importance of assaying eQTLs in diverse cell types and contexts. Does this trend hold true for the three other molecular trait eQTLs?

Thank you for the suggestion. We have now repeated the same colocalisation analysis for the other three quantification methods and found very similar results to gene expression QTLs. These new results are now presented in Supplementary Figure 9 and reference do it has been added to the caption of Figure 5.

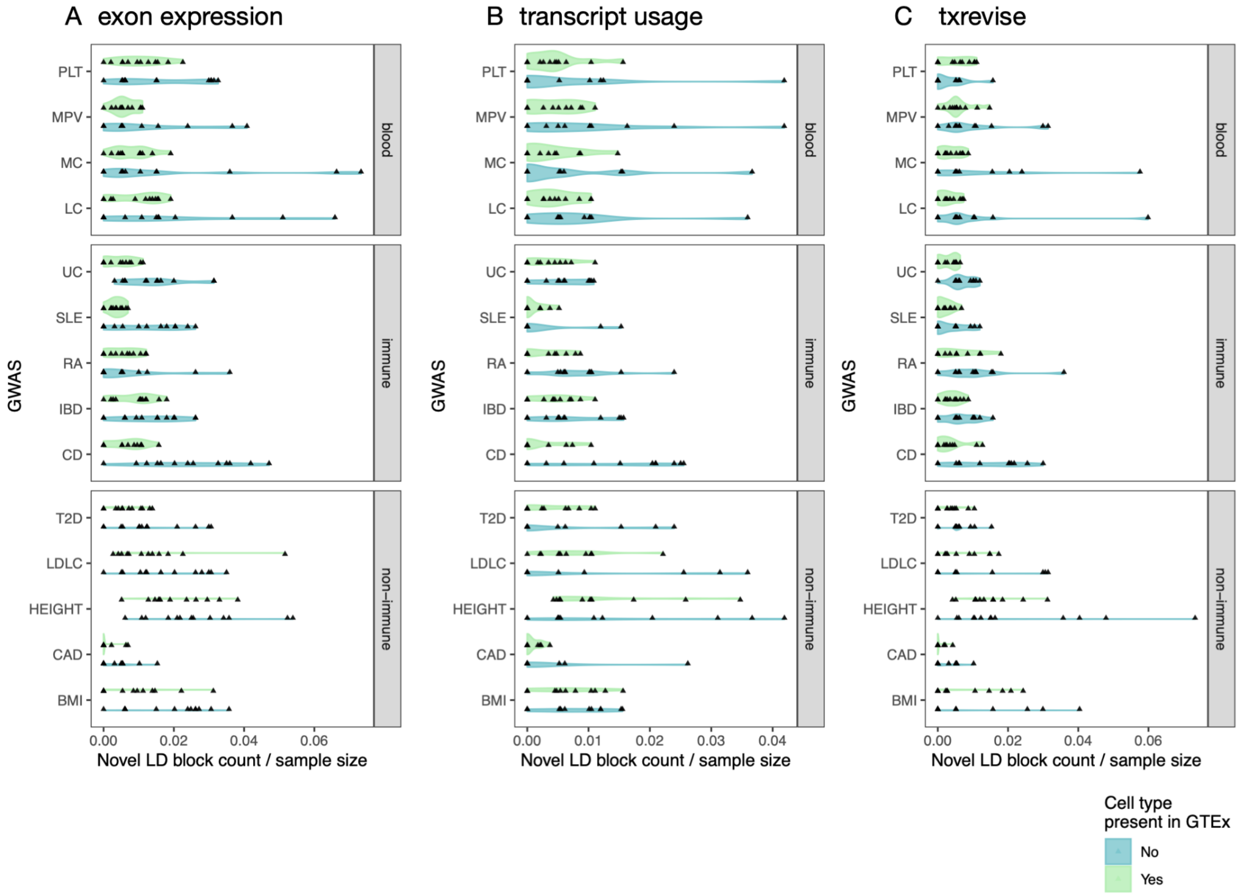

Supplementary Figure 9. The number of novel colocalising loci detected for the 14 GWAS traits in each cell type and tissue from eQTL Catalogue divided by the eQTL sample sizes. The analysis was done independently for exon QTLs (**A**), transcript usage QTLs (**B**) and txrevise QTLs (**C**). The eQTL Catalogue cell types and tissues were grouped according to whether they were present in GTEx (blood, LCL, adipose, muscle, skin, brain) or not (T cells, B cells, monocytes, macrophages, neutrophils and iPSCs). GWAS traits: PLT - platelet count, MPV - mean platelet volume, MC - monocyte count, LC - lymphocyte count, UC - ulcerative colitis, SLE

- systemic lupus erythematosus, RA - rheumatoid arthritis, IBD - inflammatory bowel disease, CD - Crohn's disease, T2D - type 2 diabetes, height, CAD - coronary artery disease, BMI - body mass index, LDLC - LDL cholesterol

8. In line 153, the mash model is introduced as "multiple adaptive shrinkage". It is actually called "multivariate adaptive shrinkage".

This has now been corrected.

Decision Letter, first revision:

Our ref: NG-A56677R

9th Jun 2021

Dear Dr. Alasoo,

Thank you for submitting your revised manuscript "eQTL Catalogue: a compendium of uniformly processed human gene expression and splicing QTLs" (NG-A56677R). It has now been seen by the original referees and their comments are below. The reviewers find that the paper has improved in revision, and therefore we'll be happy in principle to publish it in Nature Genetics, pending minor revisions to satisfy the referees' final requests and to comply with our editorial and formatting guidelines.

If the current version of your manuscript is in a PDF format, please email us a copy of the file in an editable format (Microsoft Word or LaTex)-- we can not proceed with PDFs at this stage.

We are now performing detailed checks on your paper and will send you a checklist detailing our editorial and formatting requirements in about a week. Please do not upload the final materials and make any revisions until you receive this additional information from us.

Thank you again for your interest in Nature Genetics Please do not hesitate to contact me if you have any questions.

Sincerely,

Safia Danovi
Editor
Nature Genetics

Reviewer #1 (Remarks to the Author):

The authors have addressed all my questions. The manuscript looks great.

Reviewer #2 (Remarks to the Author):

The authors have carefully addressed the comments that I raised. I therefore have no remaining comments.

Reviewer #3 (Remarks to the Author):

The authors have addressed my concerns.

Final Decision Letter:

In reply please quote: NG-A56677R1 Alasoo

26th Jul 2021

Dear Dr. Alasoo,

I am delighted to say that your manuscript "A compendium of uniformly processed human gene expression and splicing quantitative trait loci" has been accepted for publication in an upcoming issue of Nature Genetics.

Prior to setting your manuscript, we may make minor changes to enhance the lucidity of the text and with reference to our house style. We therefore ask that you examine the proofs most carefully to ensure that we have not inadvertently altered the sense of your text in any way.

Once your manuscript is typeset and you have completed the appropriate grant of rights, you will receive a link to your electronic proof via email with a request to make any corrections within 48 hours. If, when you receive your proof, you cannot meet this deadline, please inform us at rjsproduction@springernature.com immediately.

Your paper will be published online after we receive your corrections and will appear in print in the next available issue. You can find out your date of online publication by contacting the Nature Press Office (press@nature.com) after sending your e-proof corrections. Now is the time to inform your Public Relations or Press Office about your paper, as they might be interested in promoting its publication. This will allow them time to prepare an accurate and satisfactory press release. Include your manuscript tracking number (NG-A56677R1) and the name of the journal, which they will need when they contact our Press Office.

Before your paper is published online, we shall be distributing a press release to news organizations worldwide, which may very well include details of your work. We are happy for your institution or funding agency to prepare its own press release, but it must mention the embargo date and Nature Genetics. Our Press Office may contact you closer to the time of publication, but if you or your Press Office have any enquiries in the meantime, please contact press@nature.com.

Acceptance is conditional on the data in the manuscript not being published elsewhere, or announced in the print or electronic media, until the embargo/publication date. These restrictions are not intended to deter you from presenting your data at academic meetings and conferences, but any enquiries from the media about papers not yet scheduled for publication should be referred to us.

Please note that <i>Nature Genetics</i> is a Transformative Journal (TJ). Authors may publish their research with us through the traditional subscription access route or make their paper immediately open access through payment of an article-processing charge (APC). Authors will not be required to make a final decision about access to their article until it has been accepted. <a

href="https://www.springernature.com/gp/open-research/transformative-journals"> Find out more about Transformative Journals

<B>Authors may need to take specific actions to achieve compliance with funder and institutional open access mandates. For submissions from January 2021, if your research is supported by a funder that requires immediate open access (e.g. according to Plan S principles) then you should select the gold OA route, and we will direct you to the compliant route where possible. For authors selecting the subscription publication route our standard licensing terms will need to be accepted, including our self-archiving policies. Those standard licensing terms will supersede any other terms that the author or any third party may assert apply to any version of the manuscript.

Please note that Nature Research offers an immediate open access option only for papers that were first submitted after 1 January, 2021.

You will not receive your proofs until the publishing agreement has been received through our system.

If you have any questions about our publishing options, costs, Open Access requirements, or our legal forms, please contact ASJournals@springernature.com

If you have posted a preprint on any preprint server, please ensure that the preprint details are updated with a publication reference, including the DOI and a URL to the published version of the article on the journal website.

To assist our authors in disseminating their research to the broader community, our SharedIt initiative provides you with a unique shareable link that will allow anyone (with or without a subscription) to read the published article. Recipients of the link with a subscription will also be able to download and print the PDF.

As soon as your article is published, you will receive an automated email with your shareable link.

You can now use a single sign-on for all your accounts, view the status of all your manuscript submissions and reviews, access usage statistics for your published articles and download a record of your refereeing activity for the Nature journals.

An online order form for reprints of your paper is available at https://www.nature.com/reprints/author-reprints.html. Please let your coauthors and your institutions' public affairs office know that they are also welcome to order reprints by this method.

If you have not already done so, we invite you to upload the step-by-step protocols used in this manuscript to the Protocols Exchange, part of our on-line web resource, natureprotocols.com. If you complete the upload by the time you receive your manuscript proofs, we can insert links in your article that lead directly to the protocol details. Your protocol will be made freely available upon publication of

your paper. By participating in natureprotocols.com, you are enabling researchers to more readily reproduce or adapt the methodology you use. Natureprotocols.com is fully searchable, providing your protocols and paper with increased utility and visibility. Please submit your protocol to https://protocolexchange.researchsquare.com/. After entering your nature.com username and password you will need to enter your manuscript number (NG-A56677R1). Further information can be found at https://www.nature.com/nprot/.

Sincerely,

Safia Danovi
Editor
Nature Genetics
